# Transition metal oxides as a cathode for indispensable Na-ion batteries

**DOI:** 10.1039/d2ra03601k

**Published:** 2022-08-17

**Authors:** Archana Kanwade, Sheetal Gupta, Akash Kankane, Manish Kumar Tiwari, Abhishek Srivastava, Jena Akash Kumar Satrughna, Subhash Chand Yadav, Parasharam M. Shirage

**Affiliations:** Department of Metallurgy Engineering and Materials Science, Indian Institute of Technology Indore 453552 India pmshirage@iiti.ac.in paras.shirage@gmail.com; Department of Physics, Indian Institute of Technology Indore 453552 India

## Abstract

The essential requirement to harness well-known renewable energy sources like wind energy, solar energy, *etc*. as a component of an overall plan to guarantee global power sustainability will require highly efficient, high power and energy density batteries to collect the derived electrical power and balance out variations in both supply and demand. Owing to the continuous exhaustion of fossil fuels, and ever increasing ecological problems associated with global warming, there is a critical requirement for searching for an alternative energy storage technology for a better and sustainable future. Electrochemical energy storage technology could be a solution for a sustainable source of clean energy. Sodium-ion battery (SIB) technology having a complementary energy storage mechanism to the lithium-ion battery (LIB) has been attracting significant attention from the scientific community due to its abundant resources, low cost, and high energy densities. Layered transition metal oxide (TMO) based materials for SIBs could be a potential candidate for SIBs among all other cathode materials. In this paper, we discussed the latest improvement in the various structures of the layered oxide materials for SIBs. Moreover, their synthesis, overall electrochemical performance, and several challenges associated with SIBs are comprehensively discussed with a stance on future possibilities. Many articles discussed the improvement of cathode materials for SIBs, and most of them have pondered the use of Na_*x*_MO_2_ (a class of TMOs) as a possible positive electrode material for SIBs. The different phases of layered TMOs (Na_*x*_MO_2_; TM = Co, Mn, Ti, Ni, Fe, Cr, Al, V, and a combination of multiple elements) show good cycling capacity, structural stability, and Na^+^ ion conductivity, which make them promising cathode material for SIBs. This review discusses and summarizes the electrochemical redox reaction, structural transformations, significant challenges, and future prospects to improve for Na_*x*_MO_2_. Moreover, this review highlights the recent advancement of several layered TMO cathode materials for SIBs. It is expected that this review will encourage further development of layered TMOs for SIBs.

## Abstract 2

Na^+^ ion intercalated layered metal oxides have tremendous applications as the cathode materials for SIBs owing to their superior electrochemical performance compared to other types of cathode materials.

## Graphical abstract

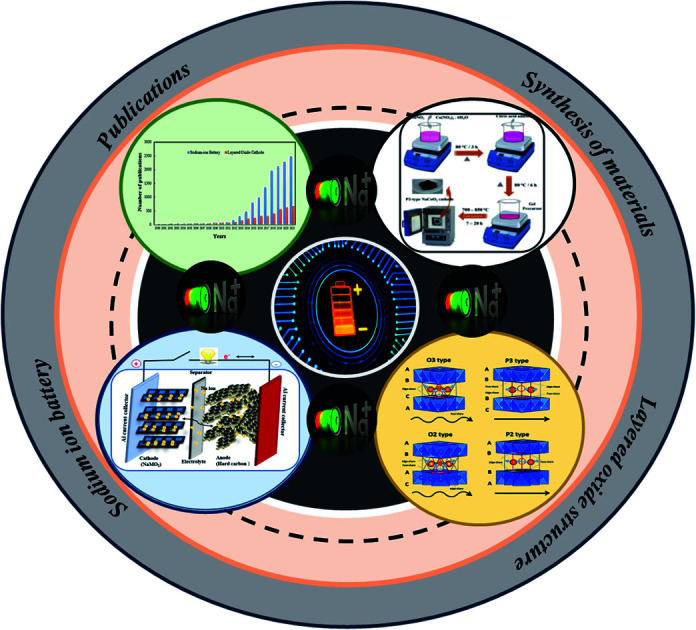

## 1. Introduction

The global energy expenditure has increased rapidly throughout the last two decades, so there is a need to develop energy storage technology with superior performance and sustainability.^1,2^ As energy appears in various forms like heat, radiation, electricity, chemistry, and gravity, energy storage methods that involve transforming energy from different forms that are challenging to collect are indispensable for the economic usage of energy.^3–5^

**Fig. 1:**
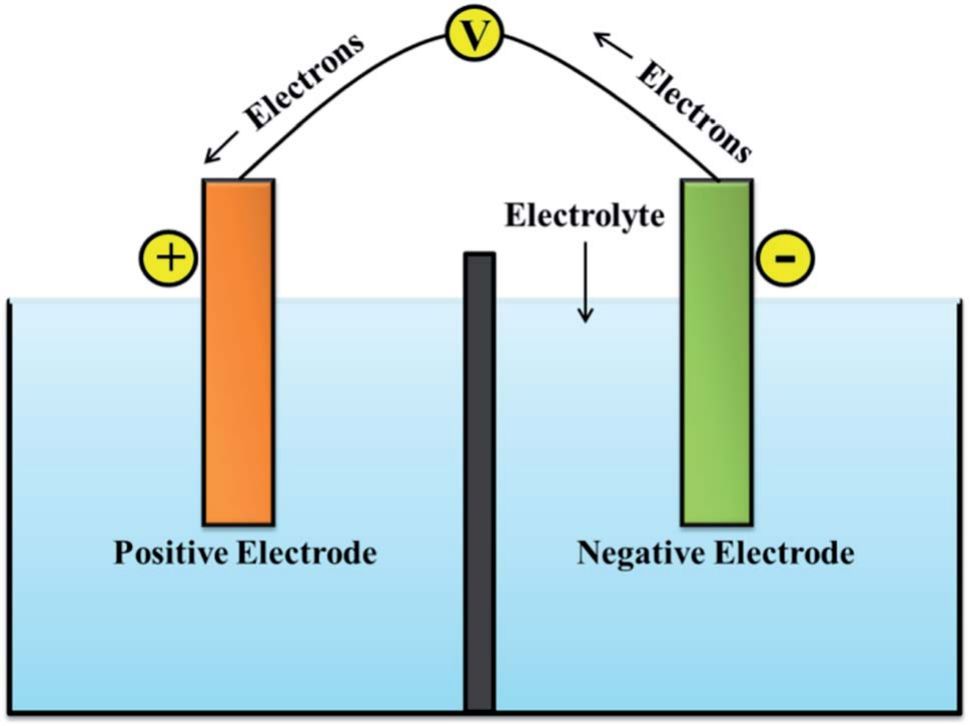
Schematic of battery system.

A battery can store electrical energy in the form of chemicals and redox reactions, and it provides direct current electricity.^6^ While discharging, the equations define a chemical reduction process by gaining electrons at the positive electrode, and an oxidation process by losing electrons at the negative electrode. The recharge of a secondary battery is a reversal of the processes that occur during discharge.^7^ The service of batteries lies in the wide variety of sizes in which they can be assembled or manufactured into packs. Their portability (for smaller sizes) and the capacity to immediately provide electrical power is the most convenient property.^8–10^ A single cell of the battery is composed of an electrolyte, a separator, a negative electrode, and a positive electrode as presented in Fig. 1.^11^

**Fig. 2:**
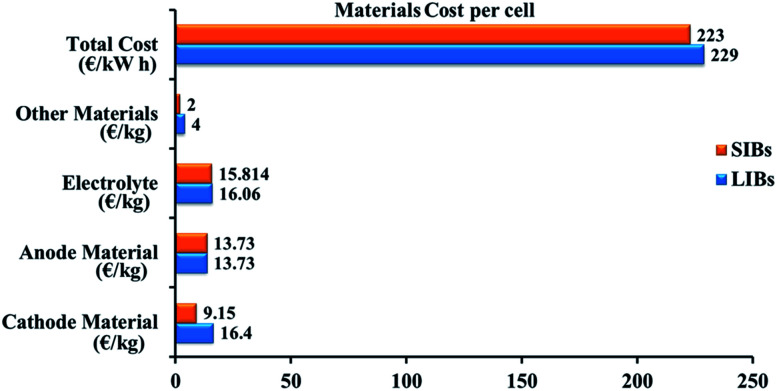
Evaluation of the fabrication costs for LIB and SIB.^21^

An electrolyte is an electronically insulating and ionically conducting source that permits the redox reaction on both electrodes. While using electrolytes in liquid form, a porous film needs to be placed as the separator separating the anode and cathode to ignore the electrical connection.^12,13^ The separator enables the liquid electrolyte to penetrate and separates the anode and cathode from contacting each other.^14^ A cell is the core component of a battery. For various compact electronic devices, a single cell can meet the power and energy demands. For large-scale utilization in electric vehicles, multiple cells are electrically integrated as modules and packed into a battery pack to satisfy the power and energy requirements.^15^ Lithium is widely used in the energy storage devices because it has a high energy density. Nevertheless, limited lithium resources are unable to fulfil the supply for large-scale utilization, particularly for the grid-scale storage of energy.^16^ Sodium is abundant in natural resources, so its price is low; sodium-ion batteries are becoming a possible replacement for lithium-ion batteries.^17^ An obvious replacement for lithium is sodium because of its analogous chemical properties.^18^ Investigations into SIBs started in the 1970s along with LIBs, but real research work only began after Sony was successful marketing LIBs in 1990.^19^ Despite the higher ionic radius that sodium (1.02 Å) has over lithium (0.76 Å), the cost per kW h of energy that sodium provide is less than the lithium can provide. The cost of fabrication of LIB and SIB is represented Fig. 2. A brief comparison between lithium and sodium can be seen in Table 1.^20^

**Table 1:** Characteristics of lithium *vs.* sodium

Category	Lithium	Sodium
Atomic weight	6.9 g mol^−1^	23 g mol^−1^
Cation radius	0.76 Å	1.02 Å
Cost	High	Low
Preferred coordination	Tetrahedral and octahedral	Prismatic and octahedral
Capacity (mA h g^−1^)	3829	1165
*E* ^0^ (*vs.* Li/Li^+^)	0	0.3 V

It is worthy to note that SIBs had also been considered in the early days of lithium-ion battery research.^22^ The analysis, however, was largely interrupted, most likely due to slow progress and the successful chemistry of LIB.^23^ Today, the main inspiration for the research on SIBs is the abundance (less prone to resource issues) of sodium and the expectation is to manufacture cheap batteries compared to LIBs. Gross performance and the entire cost of SIBs are depends on one of the key components, cathode material. Still, it is difficult to stabilize the structure and enhance the electrochemical performance of the cathode material to satisfy the requirements of the practical application.^24,25^ Based on the chemical replacement of the prototypical cathode, LiCoO_2_ the structure of cathode materials in most of the best performing lithium-ion batteries is layered two-dimensional crystallographic.^26^

**Fig. 3:**
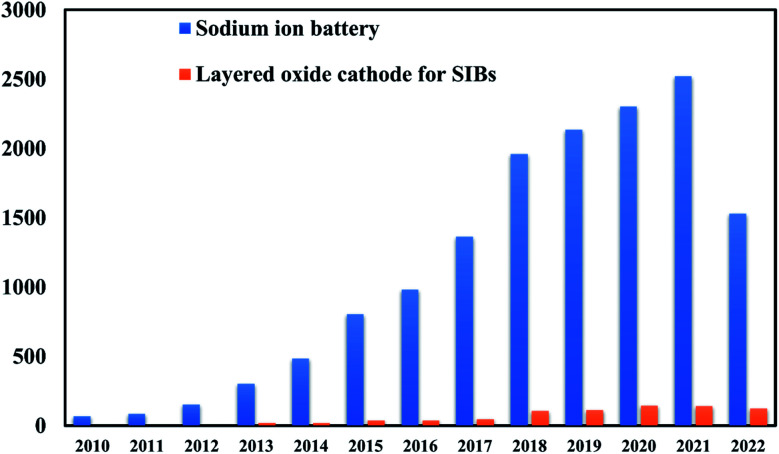
Graphical representation of number of scientific publications on Na-ion battery and layered cathode materials every year from 2000 to 2021. (Data are encapsulated from Scopus).

Fig. 3 represents the number of scientific publications on Na-ion battery, and layered oxide cathodes. The key challenges for commercialization of SIBs are the limited cycle life and low energy density of electrode materials.^27^ Cathode is mainly valuable and significantly controls the cycle life and energy density. It is challenging to simply replicate a cathode from its lithium equivalent to manufacture electrodes for SIBs due to the larger ionic radius of Na^+^ and marginally different chemistry between Li^+^ ions and Na^+^ ions.^28^ The cathode material for the most promising SIB has the same but rich, multilayered geometry. It has been well recognized that the electrochemical performance of an electrode material is strongly connected to its intrinsic crystal structure.^29^ Layered transition metal oxides are one of the best options available for the cathode of SIBs. Various properties like cyclic number and capacity of the SIBs depends on structure of layered oxide material. The stacking arrangements of layered TMOs was demonstrated by Delmas and Hagenmuller.^30^

**Fig. 4:**
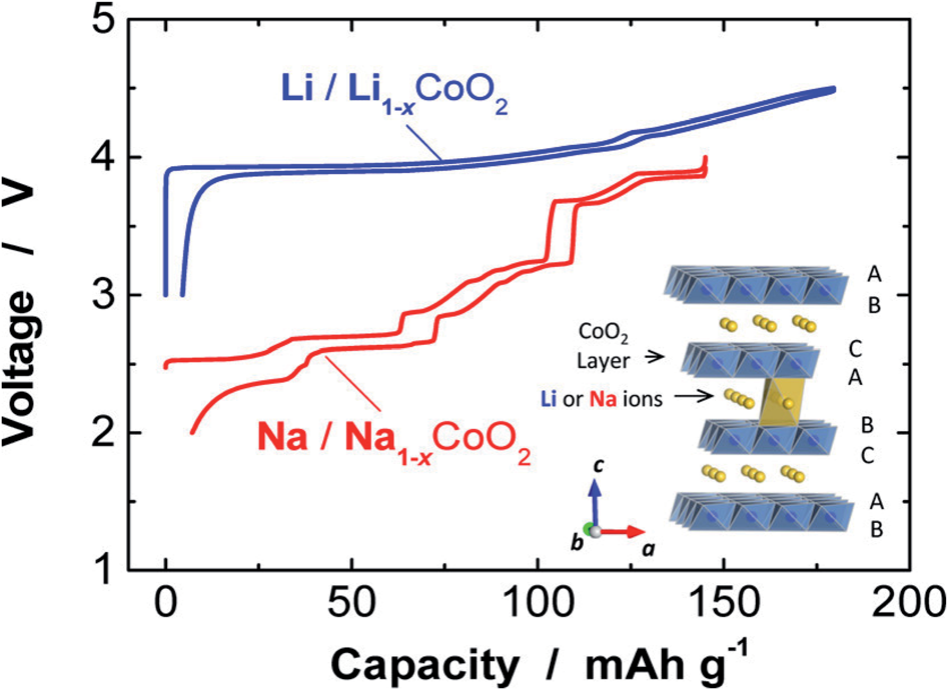
Comparison of charge/discharge plots of Na/NaCoO_2_ and Li/LiCoO_2_ cells. (Adapted from ref. 31, copyright 2014 American Chemical Society).

Fig. 4 shows the comparison of charge/discharge plots of Li/LiCoO_2_ and Na/NaCoO_2_ cells. A larger ionic radius of the sodium ion is the result of an extra structural richness, which has an extended octahedral geometry determined in the cathode material of Li-ion. The coordination environment of the alkali metal is denoted by either O for octahedral or P for prismatic coordination, followed by a number denoting the layers of transition metal in the stacking repeat unit. The large MO_6_ sheets gives 2D transport channels for the insertion/extraction of Na^+^ ions in between them, offering high specific capacity to Na_*x*_MO_2_.^32^ After crystallization, its configuration is trigonal prismatic. This additional versatility of structural offers the possibility to modify for energy and power applications. It suggests the direction of research for better performance of SIB that comprises higher discharge and charge rates, enhanced capacity, increased cyclability, better recycling, and long lifetimes. Efficient charge storage and transfer set the condition for stable handling of an electrochemical energy storage device, because of their excellent structural features, such as decreased particle dimensions and large surface-to-volume fraction, nanomaterials hold commitment in promoting storage kinetics and allowing the unique storage chemistry of electrode substances.^33^ LIBs have reshaped the world of digital electronics and provided the future electrification of domestic and transport energy storage. SIBs can expand and extend this possibility to provide environmentally sustainable and safer options. Recent important developments show that SIBs can attain similar performances as LIBs. Further discovery regarding the energy density of SIBs depends on the improvement of high-performance material for electrode.^34^ SIBs usually have one negative and one positive electrode, the separator, an electrolyte, and the case of the battery. Moreover, sodium salts are plentiful in nature and non-toxic, easy to extract, and cheap.

## 2. Components of battery

There are three main components of battery: electrodes, electrolyte, and separator. They are explained briefly and shown in Table 2.

**Table 2:** Components of LIBs and SIBs^a^

Chemistry	SIB	LIB
Anode	Hard carbon on foil of aluminium, organic binder	Graphite on foil of copper, organic or aqueous binder
Cathode	Layered oxide on foil of aluminium, organic binder	Layered oxide foil of aluminium, organic binder
Separator	Polymer film (mostly PE)	Polymer film (mostly PE)
Electrolyte	Na salt (NaPF_6_, NaClO_4_)in organic solvent (EC/DMC)	Li salt (LiPF_6_, LiTFSI) in organic solvent (EC/DMC)
Cell housing	Prismatic, pouch, or round cells	Pouch, prismatic, or round cells

a EC = ethylene carbonate, PE = polyethylene, DMC = dimethyl carbonate.

### 2.1 Electrodes

Anode and cathode are the two electrodes which contribute to the storage of energy. Anode is the electrode of a battery that loses electrons and cathode is the electrode that gains electrons while discharging. These electrodes should have ionically as well as electronically conductive and chemically compatible with the electrolyte. There are different types of cathode materials for SIBs which are discussed in detail in below section. Also, the anode materials can be categorized into carbons, metals and alloys, low potential TMOs and phosphates.

### 2.2 Electrolyte

Electrolyte is the medium between the two electrode polarities of a cell that provides ionic conductivity. Electrolytes are an essential element of all electrochemical devices. In most electrochemical devices such as batteries, aqueous solutions of acids and salts are used as electrolytes. Aqueous solutions are convenient, easy to prepare, and should be made from relatively inexpensive and readily available materials. If the electrolyte has electronic conductivity, it can give rise to self-discharge, and an internal short-circuit would occur within the cell.^35^ When there is a surplus of liquid electrolytes, the term used is the flooded-electrolyte battery. Still, if the liquid electrolyte is fully immobilized within the separator, it is referred to a starved-electrolyte battery.^36^ Not only lead-acid batteries but also vanadium redox flow batteries and Zn/Br batteries uses active electrolyte which takes part in the half-cell reactions.^37^ Most batteries, especially those with an aqueous electrolyte, undergo slow self-discharge when left standing on an open circuit. Other ionic conductors like fused salts, polymers, organic salt solutions, and ceramics are used as electrolytes. Purification of the electrolyte solutions is crucial, especially concerning separating the last traces of water.^38^ Electrolyte works as a path provider for positive ions moving from cathode to anode while charging and that from anode to cathode while discharging. Ions are electrically charged atoms that have gained or lost electrons.

The electrolyte should have zero electronic and high ionic conductivity to prevent short-circuiting of the electrons, high thermodynamic stability in a wide voltage and temperature range, and high chemical and physical compatibility with anode and cathode.

### 2.3 Separator

The separator is an essential component of the battery that separates the negative electrode and positive electrode in the battery to prevent the negative and positive electrodes from direct contact, which may cause a short circuit.^39^ It has a porous structure to give a passage for the ions, realizing ions transportation between the positive and negative electrodes. The separator has no participation in the battery's reaction, although it shows a crucial role in the battery. The separator influences safety performance, battery capacity, cycle performance, and rate performance to some extent.^40^ Currently, non-woven fabric separators and polyolefin separators are generally utilized commercially. The chief factors of the separator involve mechanical strength, chemical stability, wettability, heat resistance, and porosity.^34^ Analyzing the earlier factors to choose the suitable separator material, rechargeable battery separators are principally classified into polyolefin-based separators, ceramic composite separators, and non-woven separators. Various formation processes and the replacement of the separator's function have been directed to some latest types of separators, like polymer electrolyte separators.

## 3. Working of sodium ion battery

**Fig. 5:**
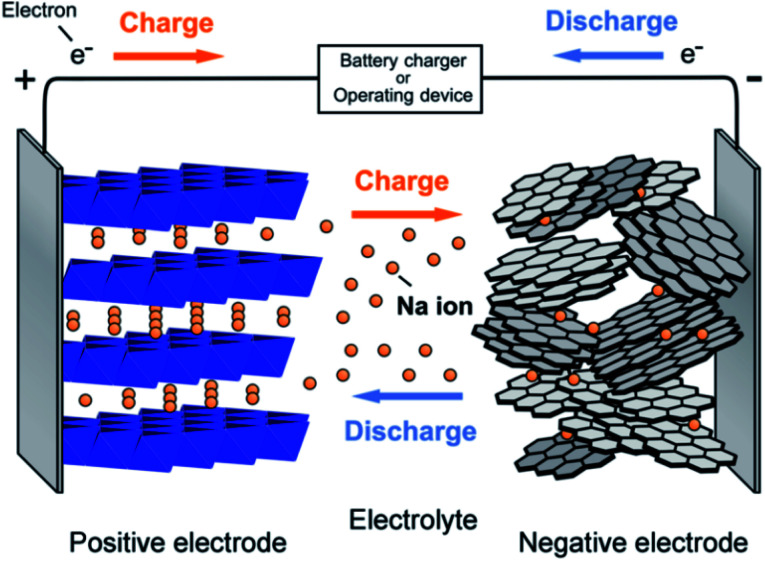
Schematic showing the working principle of the sodium ion battery. (Adapted from ref. 31, copyright 2014 American Chemical Society).

In charging, sodium ions are extracted from the positive electrode and passed by an electrolyte, reached into the negative electrode. When the current flow in an external circuit, the battery discharges spontaneously, *i.e.*, on the anode, the oxidation reaction occurs, related to the sodium ions departs from the structure of the negative electrode and reached into the layers of the cathode. A rechargeable Na^+^ ion cell consists of two materials capable of Na^+^ ion insertion called negative electrode and positive electrode. The electrolyte (pure ionic conductor) electronically isolates these electrodes. The stored chemical energy is converted into electrical energy, and the performance of the entire assembly changes with varying the shape, state, composition, alignment of these components. A representation of the operating principle of SIBs is shown in Fig. 5.

In secondary battery technologies, when sodium replaces lithium, it requires completely new anode materials. The most remarkably suitable anode material, graphite in LIB, is incapable of intercalating sodium metal ions between its nanosheets. Hard carbon or no graphitizable carbon, which can intercalate sodium ions are used an anode material in SIBs.^41–43^ The overall chemical reaction for charging and discharging of NaMnO_2_ is shown below:

While charging,At cathode: NaMnO_2_ → Na_1−*x*_MnO_2_ + *x*Na^+^ + *x*e^−^At anode: C + *x*Na^+^ + *x*e^−^ → Na_*x*_COverall: C + NaMnO_2_ → Na_1−*x*_MnO_2_ + Na_*x*_C

While discharging,At cathode: Na_1−*x*_MnO_2_ + *x*Na^+^ + *x*e^−^ → NaMnO_2_At anode: Na_*x*_C → C + *x*Na^+^ + *x*e^−^Overall: Na_1−*x*_MnO_2_ + Na_*x*_C → NaMnO_2_ + C

## 4. Cathode materials for SIBs

**Fig. 6:**
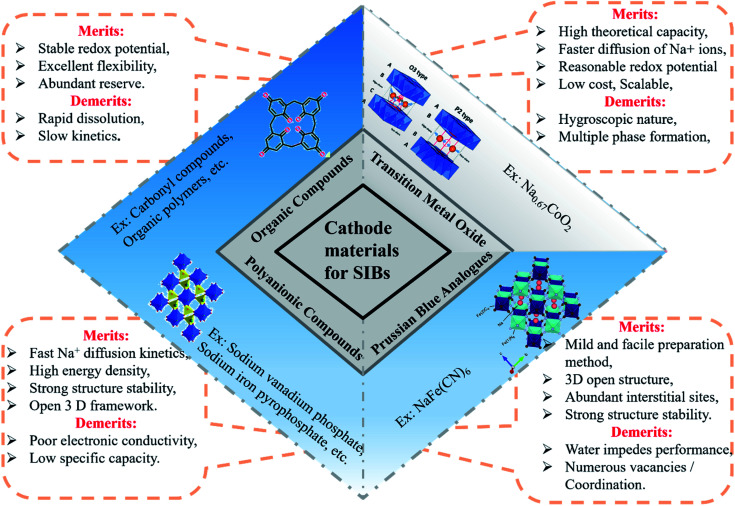
Schematic illustration of types of cathode materials for SIB.

**Fig. 7:**
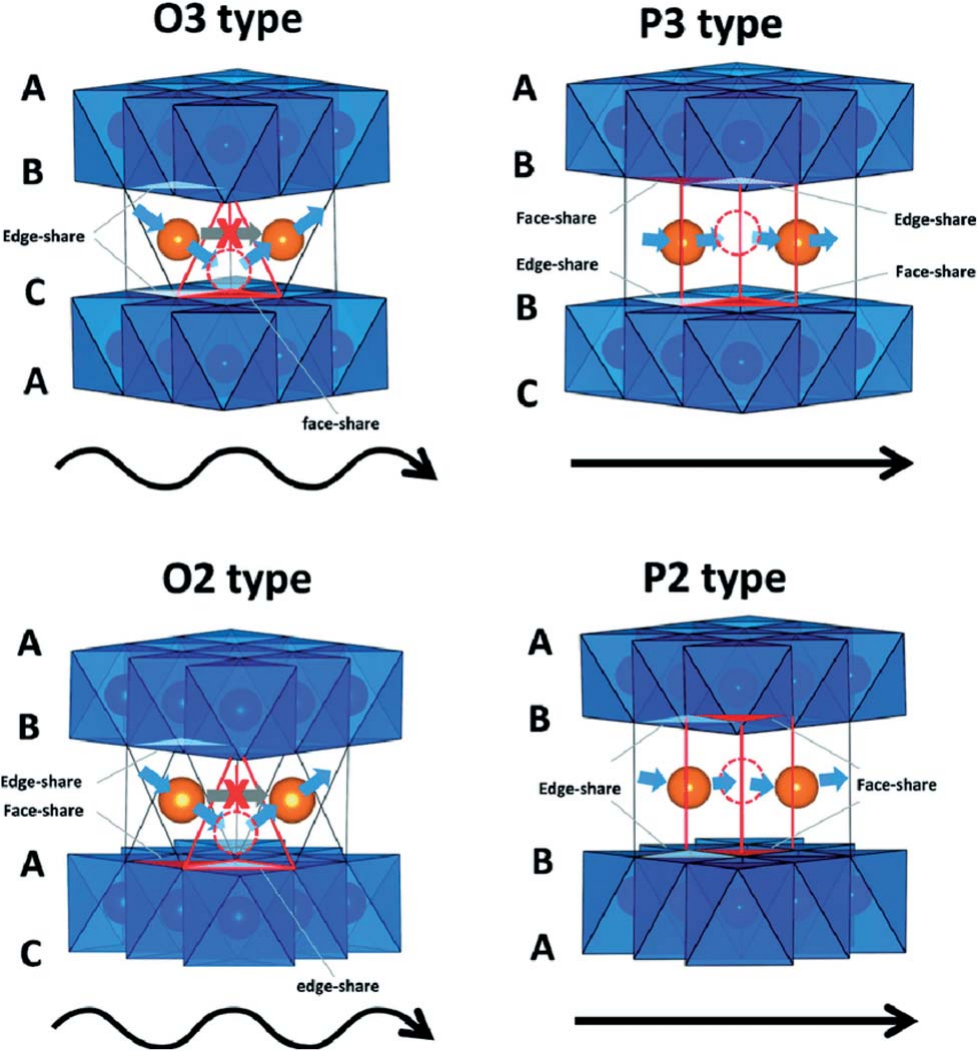
Na^+^ migration paths in different layered structures. (Adapted from ref. 31, copyright 2014 American Chemical Society).

The cathode materials for SIBs are categorized into four types *viz.* layered transition metal oxides,^44^ polyanionic compounds,^45^ Prussian blue (PB) analogues and organic compounds as shown in Fig. 6. PBs are deficient for Na^+^ storage because of the vacancies present. During process of de/intercalation, Fe–C≡N–Fe bond disrupts which results in lattice distortion. It is a challenge to synthesize PBs at room temperature having high efficiency.^46–48^ Polyanionic cathode materials provides better safety and they are non-hygroscopic in nature but they exhibit poor cycling performance.^49^ Layered transition metal oxides (TMOs) Na_*x*_MO_2_ (here, M = transition metals, can be single or multiple metal elements) are expected to be promising cathode materials for SIBs. The specific structure has small ion diffusion channels and a bigger surface area during sodiation/desodiation that are advantageous for stabilizing cycling capacity.^50–53^ The prosperity of LiCoO_2_ unsurprisingly inspires research into its sodium equivalent, NaCoO_2_. Layered sodium oxide materials can be categorized based on the position of Na^+^ ions. The terminology for these materials is as follows: P and O refer to Na's trigonal and octahedral coordination, and the number 3 or 2 refers to the repetition of layers of transition metal in the unit cell. For example, in O3, it's ABCABC, and in P2, it's ABBA, as illustrated in Fig. 7. The withdrawal of sodium ions from O3 and P2-type phases of NaMO_2_ (M = Fe, Ni, Co, Mn) compounds mostly drives phase transitions.^54,55^

**Fig. 8:**
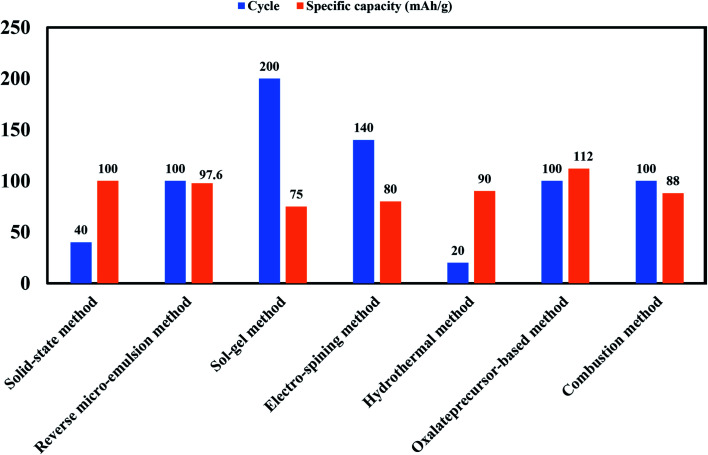
Bar chart showing comparison of specific capacity and cycle number for material Na_0.44_MnO_2_ made by various processes as cathodes for sodium-ion batteries.

Comparison of specific capacity and cycle number for material Na_0.44_MnO_2_ made by various processes as cathodes for sodium-ion batteries presented in Fig. 8. Although the highest energy density can be determined for Li-ion cathodes, various Na ion cathodes showing values that make them prospective candidates. The graphical representation also spotlights the obstacle of finding improved Na-ion cathodes. TMOs normally possess high reversible capacities. The compact structural framework of metal oxides makes them ideally appropriate for applications that requires a high volumetric density.^56^

### 4.1 O3-type transition metal oxides

Edge-shared octahedral sites are the initially stabilized site for sodium ions in O3-type structures. Prismatic positions get energetically stable when Na^+^ ions are partially pulled out from the O3 phase. The gliding of layers of MO_2_ can accomplish this without breaking M–O bonds. P3-type material can be formed by varying the packing of oxygen in the structure. The transition between the P3/O3-type and P2-type phase is not possible in Na cells and can only be attained by reforming and breaking M–O bonds in a high-temperature surrounding. At the time of the sodium intercalation process, the O3 phase always transforms to the P3 phase. Generally, O3-type materials have more Na content than P2-phase materials.^57^ O3 based systems are associated with the best battery capacity performances that have the higher sodium stoichiometry.^58^

Kubota, Kei *et al.* reported effect of Ti and Mg doping in NaNi_1/2_Mn_1/2_O_2_ (O3 type layered oxide) on phase transition and reversibility during electrochemical intercalation of sodium ions. The structure of NaNi_1/2_Mn_1/2_O_2_ is O3 type layered oxide and it is same as α-NaFeO_2_, has drawn interest as a cathode material for SIBs due to its larger reversible capacity of 200 mA h g^−1^.^59^ The O3 type NaNi_1/2_Mn_1/2_O_2_ materials with Ti or Mg substitution show good capacity, and co-substituted Ti and Mg material shows a discharge capacity of 200 mA h g^−1^ with no loss of capacity owing to substitution. Replacement of Ti^4+^ and Mg^2+^, (bigger ions than Mn^4+^ or Ni^2+^), outcomes a greater in-plane lattice of the O3 type structure, this could postpone the transition of phase in charging. The coexistence of Ti and Mg improves the reversibility and the structural stability at the surface of material, rising in the performance of SIB.^60,61^

**Fig. 9:**
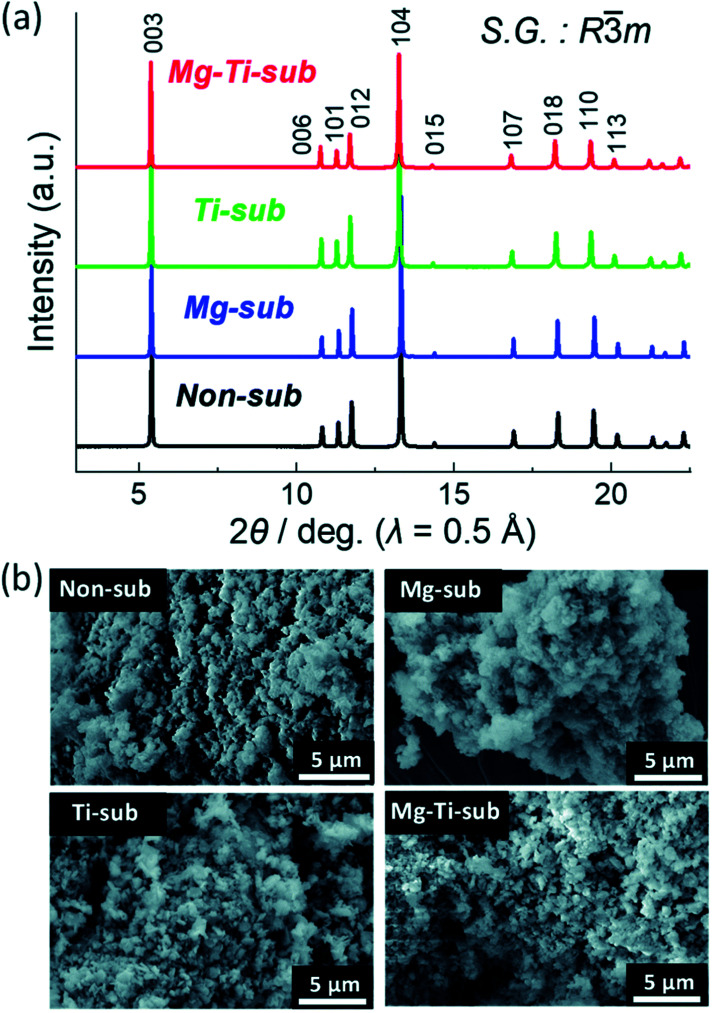
(a) X-ray diffraction patterns and (b) SEM images of pristine Na[Ni_4/9_Mn_1/3_Mg_1/18_Ti_1/6_]O_2_ (Mg–Ti-sub), Na[Ni_1/2_Mn_1/2_]O_2_ (non-sub), Na[Ni_4/9_Mn_1/2_Mg_1/18_]O_2_ (Mg-sub) and Na[Ni_1/2_Mn_1/3_Ti_1/6_]O_2_ (Ti-sub). (Adapted from ref. 59, copyright 2021 Royal Society of Chemistry).

**Fig. 10:**
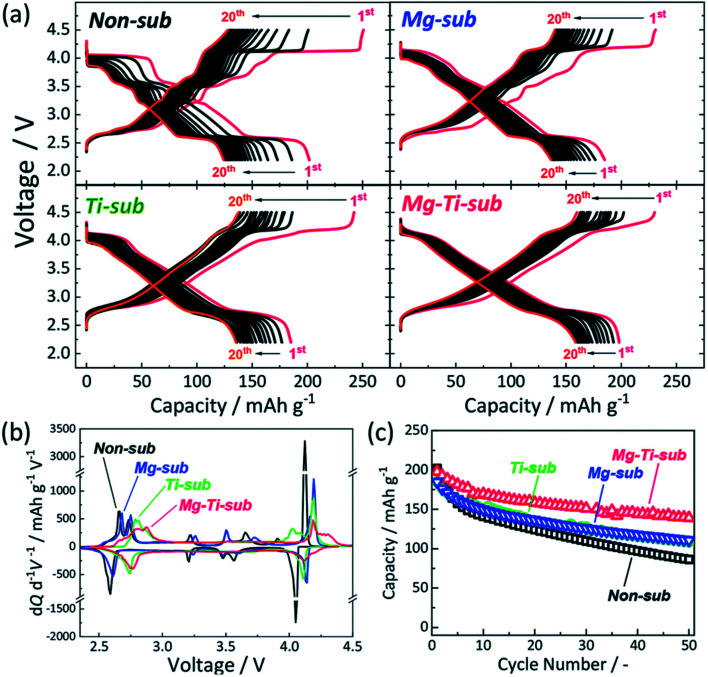
(a) Galvanometric charge–discharge plot, (b) d*Q*/d*V* plot, and (c) cyclic stability of Mg–Ti-sub, non-sub, Mg-sub and Ti-sub and electrodes in Na-ion cells. At 12 mA g^−1^ current density galvanostatic charge–discharge experiments were conducted. (Adapted from ref. 60, copyright 2021 Royal Society of Chemistry).

The samples of Ti-sub, Ti–Mg-sub, Mg-sub and non-sub (sub for substituted) are shown in Fig. 9(a) using powder synchrotron XRD. For layered oxide phases of the O3 type and *R*3̄*m* space group, indexing of the Bragg's diffraction peaks is carried out. SEM was used to examine the particle's morphology, as shown in Fig. 9(b). All the samples exhibit nearly the similar morphological characterization. Particles of about hundred nanometres in diameter are homogeneously accumulated. Fig. 10(a) demonstrates galvanostatic discharge/charge plot in the broad voltage limit (2.2–4.5 V) for the initial 20 cycles at 12 mA g^−1^ current density. Ti-substituted and Mg-substituted electrodes give marginally reduced discharge capacities of 185 mA h g^−1^ compared to 202 mA h g^−1^ for non-substituted at the first cycle. Ti–Mg-substituted provides a high reversible capacity (198 mA h g^−1^), that is nearly equal to non-substituted. Ti- or Mg-substituted materials provide marginally lesser reversible capacities (185 mA h g^−1^) and show better capacity in non-aqueous Na cells than non-substituted. The Ti and Mg co-substituted material shows a larger first discharge capacity (200 mA h g^−1^).^60^ As reported by Kubota Kei *et al.* the initial coulombic efficiency of Mg-substituted and non-substituted electrodes shows nearly the similar value (80%) but Ti-substituted electrode displays marginally reduced efficiency (76%). Ti–Mg-substituted electrode shows the maximum efficiency of 86%. Ti–Mg-substituted and Ti-substituted shows sloping voltage profiles while Mg-substituted and non-substituted samples displays stepwise voltage profiles which are clearly examined as redox peaks in the differential plots in Fig. 10(b). Ti-substituted and Mg-substituted electrodes shows enhanced cyclic stability for 50 cycles with 60% of the first discharge capacities that is 27% more than non-substituted. The co-substitution shows improved cyclic performance. At the 50th cycle, the Ti–Mg-substituted electrode gives 70% of the first discharge capacity. Similar substantial development is detected for Ti and Cu co-substituted^62–64^ and Ti and Zn co-substituted Na[Ni_1/2_Mn_1/2_]O_2_.^65^ Therefore, dual substitution with a divalent metal ion and a tetravalent titanium ion, that contains not only Zn^2+^ and Cu^2+^ ions but also Mg^2+^ ion, is more effective in refining the electrochemical characteristics of Na[Ni_1/2_Mn_1/2_]O_2_ and meaningly improves cycle stability even in the region of high potential (>4.0 volts *vs.* Na^+^/Na). The outcomes informed by Kubota Kei *et al.* suggest that Ti and Mg co-substitution stabilizes layered oxide structures in both surface and bulk. Mg-substitution would be helpful as a HF scavenger, defending the particle surface to counter the harm of electrolyte.^66^ The replacement only of Mg^2+^ and only of Ti^4+^ results in small enhancement of cycle stability Fig. 10(c).

**Fig. 11:**
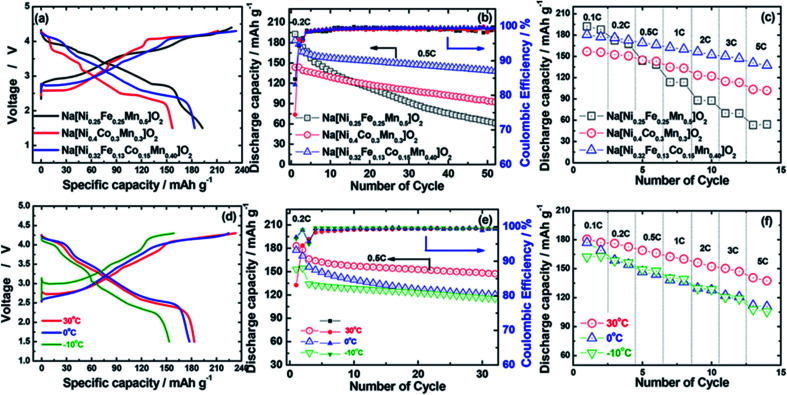
(a) First charge–discharge curves at 0.2C rate (30 mA g^−1^), (b) cycle life test at 0.5C rate (75 mA g^−1^), and (c) rate capabilities test of Na[Ni_0.25_Fe_0.25_Mn_0.5_]O_2_, Na[Ni_0.4_Co_0.3_Mn_0.3_]O_2_, and Na[Ni_0.32_Fe_0.13_Co_0.15_Mn_0.40_]O_2_ cathodes in the voltage range of 1.5–4.3 V at 30 °C. (d) First charge–discharge curves at 0.2C rate (30 mA g^−1^), (e) cycle life test at 0.5C rate (75 mA g^−1^), and (f) rate capabilities of Na[Ni_0.32_Fe_0.13_Co_0.15_Mn_0.40_]O_2_ cathodes at various operating temperatures from −10 to 30 °C. For a rate capability test, all cells were charged to 4.3 V with a constant C rate of 0.1C (15 mA g^−1^) and then discharged to 1.5 V with a different C rate ranging from 0.1C (15 mA g^−1^) to 5C (750 mA g^−1^). (Adapted from ref. 67, copyright 2018 American Chemical Society).

Hwang *et al.* synthesized O3-type Na[Ni_0.32_Co_0.13_Fe_0.15_Mn_0.4_]O_2_ cathode material *via* coprecipitation method. The various characterizations revealed that quaternary transition metal Fe substitution helped to enhance the specific capacity. Quaternary TMO frameworks effectively avoid the dissolution of transition metal during cycling. This compound displayed higher specific capacity with excellent cycling stability.^67^ This cathode material displayed the good electrochemical properties as shown in Fig. 11.

The other transition metals such as Ti and Zr maximize the electronic delocalization and entropy of mixing resulting in the improved structural stability. This type of doping elements enhances the electrochemical properties in terms of reversible specific capacity ∼141.4 mA h g^−1^.^68^ It is concluded from recent studies that the doping and substitution of transition elements to the TMOs effectively improves the performance of the SIBs.^69–71^

### 4.2 P2-type transition metal oxides

Layered P2-type compounds have exhibited increased electrochemical properties as, in general, they experience fewer phase transitions when de/intercalating Na^+^ ions than O3-type compounds.^73^ For the P2 phase, it is much easier to maintain its original structure. The electrochemical results suggest that the P2 phase shows better cycling stability and rate capability than the P3 phase.^74^Fig. 7 shows Na^+^ migration paths in different layered structures. Many O3 and P2 layered materials have been examined as they are potentially used as the cathode. O and P, respectively refer the coordination of elongated octahedral and trigonal prismatic sites of Na-ion between sheets of metal-oxide (MO_2_). Assuming stacking of the sheets normal to *Z*-direction, three possible positions can be occupied by the oxygen atoms in the *XY* plane of each layer, designated A, B, and C. All the MO_2_ sheet has two layers of oxygen atoms which is paired below and above the metal layer.^75^

**Fig. 12:**
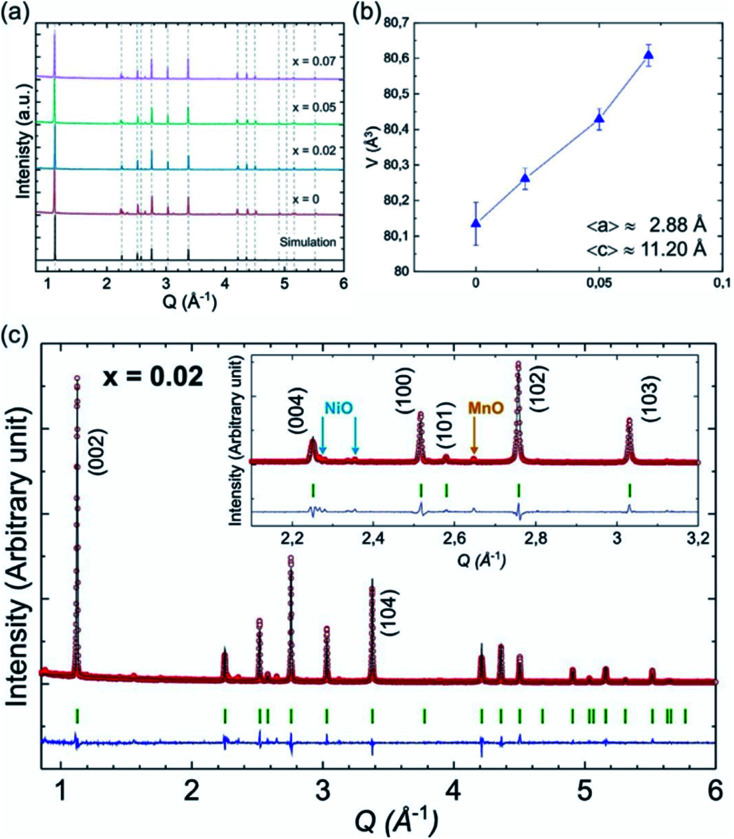
(a) XRD pattern of P2-type Na_0.5_Mn_0.83_Mg_*x*_Ni_0.17−*x*_O_2_ (*x* = 0.07, 0.05, 0.02 and 0) samples together with a simulated pattern based on *x* = 0 structure. (b) Cell volume for different Mg content (*x*) along with the average lattice parameters 〈*a*〉 and 〈*c*〉. (c) Rietveld refinement of the XRD data for the *x* = 0.02 sample. (Adapted from ref. 72, copyright 2021 Royal Society of Chemistry).

**Fig. 13:**
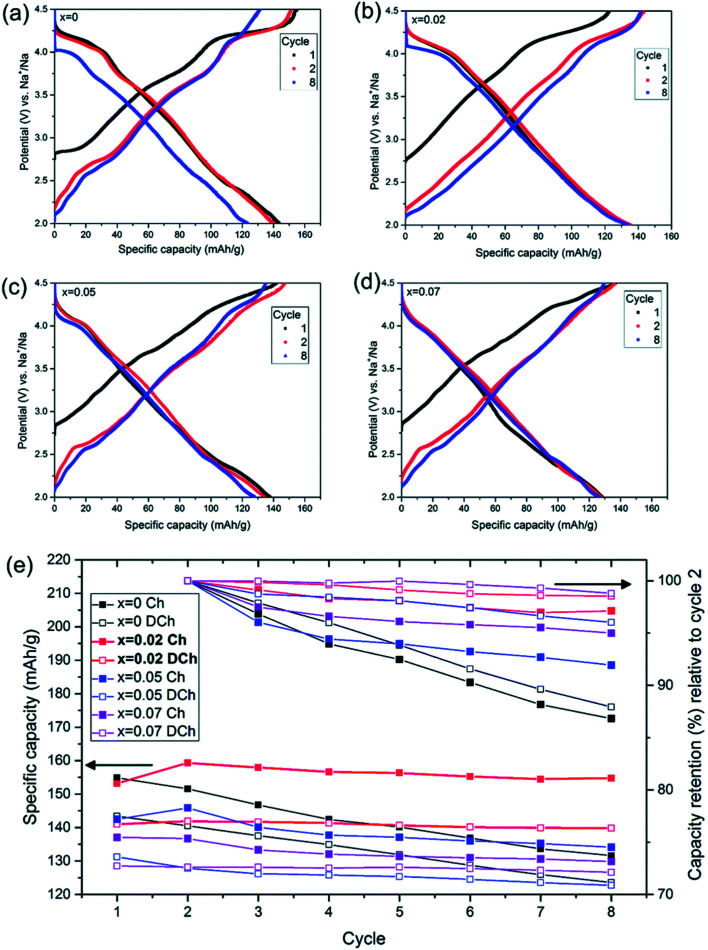
Galvanostatic charge discharge examined at 0.1C in the voltage of 2–4.5 V for P2–Na_0.5_Mg_*x*_Ni_0.17−*x*_Mn_0.87_O_2_ by using NaPF_6_ in PC as the electrolyte with various Mg contents (*x* = 0–0.07). Galvanostatic charge/discharge plot of (a) Na_0.5_Ni_0.17_Mn_0.87_O_2_, (b) Na_0.5_Mg_0.02_Ni_0.15_Mn_0.87_O_2_, (c) Na_0.5_Mg_0.05_Ni_0.12_Mn_0.87_O_2_ and (d) Na_0.5_Mg_0.07_Ni_0.10_Mn_0.87_O_2_. (e) Cycle number *vs.* capacity. (Adapted from ref. 72, copyright 2021 Royal Society of Chemistry).

Palm, R. *et al.* synthesized polycrystalline Na_0.5_Mg_*x*_Ni_0.17−*x*_Mn_0.83_O_2_ (*x* = 0.07, 0.05, 0.02 and 0) *via* solid state method. The structure of crystal and quality of samples were validated using XRD analysis in Fig. 12. Fig. 13 displays the galvanostatic characteristics of the various samples examined at 0.1C in the voltage limit of 2–4.5 V. Fig. 13(a) displays undoped P2–Na_0.5_Ni_0.17_Mn_0.87_O_2_, three peaks at 4.2 V, 3.7 V and 2.8 V. The initial charge capacity was 150 mA h g^−1^, equivalent to 0.55 sodium extraction hypothetically, that is higher than the initial content of sodium from the chemical formula.^76^ Various reasons describe the extra capacity: at high value of voltages, the sample suffers a conversion of phase from P2 type to the O2 type phase,^77^ yielding high capacity; due to the oxygen redox contribution the additional capacity is produced,^78–81^ and electrolyte decay at high range of voltage.^82^ With continuous cycling, the peak above 4.2 V vanished, which specifies procedures at high value of voltages decline because of their instability and irreversibility. Fig. 13(b)–(d) displays the galvanostatic cycling performance of the samples with several doping of Mg, *x* = 0.07, 0.05 and 0.02. Fig. 13(e) illustrates an evaluation of the specific capacity and cyclability. Usually, all Mg-doped materials have a better retention of capacity compared to the sample without doping, that is because of the stable development of the reversible OP4 phase at elevated voltages than with the permanent O2 phase development in the material with no doping.^81,83^ The doping *x* = 0.02 shows the lowermost fading of capacity, while also demonstrating the maximum specific capacity among all samples. Various studies show even a minute Mg replacement has a powerful impact on the gross performance of P2-phase Na_0.5_Mg_*x*_Ni_0.17−*x*_Mn_0.83_O_2_. Particularly, the Mg replacement of *x* = 0.02 is the highly favourable configuration because of its maximum redox reversibility because of the highest differences in potential, highest capacity retention and the maximum mobility of Na-ion. By Rietveld refinement of the XRD data for the *x* = 0.02 sample, it can be observed that minor impurity phases (≤5%) most likely corresponding to nickel and manganese oxide compounds, which could partially come from unreacted synthesis starting materials (NiO and MnO). The starting materials are marked by blue (NiO, hexagonal) and orange (MnO, hexagonal) arrows, respectively and represent possible compounds.

**Fig. 14:**
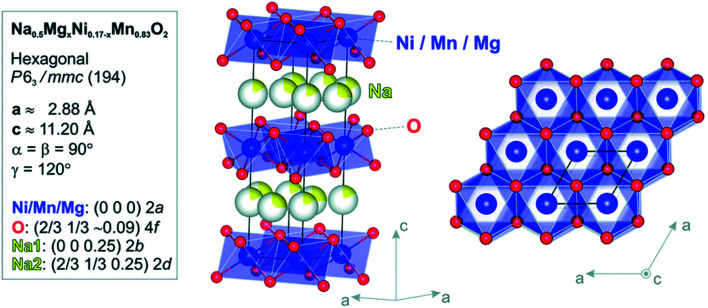
Model of atomic structure (side-view and top-view) and the atomic positions and crystallographic constraints for Na_0.5_Mn_0.83_Mg_*x*_Ni_0.17−*x*_O_2_ calculated by Rietveld refinement study of the RTXRD pattern based on the structure of *x* = 0 sample. (Adapted from ref. 72, copyright 2021 Royal Society of Chemistry).

In Fig. 14 the Ni atoms will be replaced by Mg atom into the 2a lattice sites. The fractional (2d) site occupancy is represented by the yellow partial colouring of the Na atom. Also, the existence of two different sites: Na2 (2d) and Na1 (2b) are verified.

**Fig. 15:**
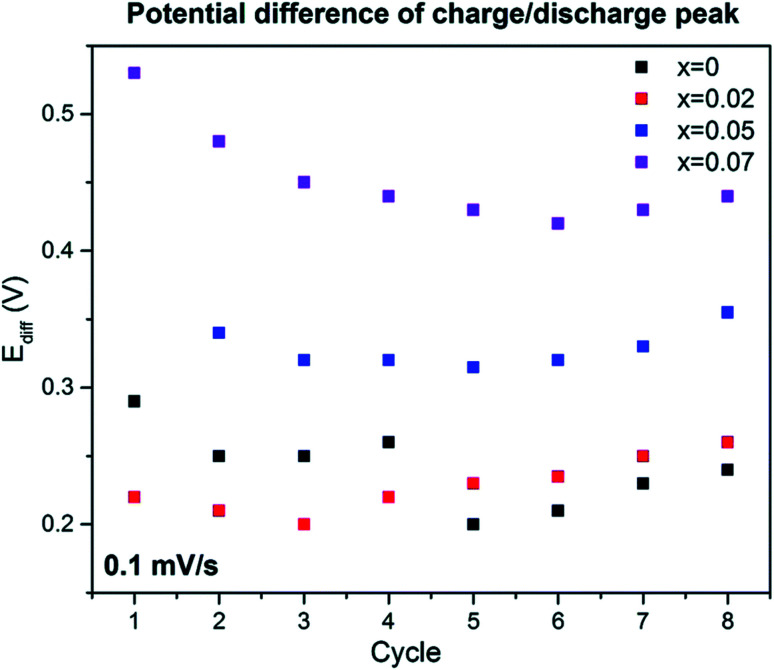
Inspection from cyclic voltammetry data at scan rate 0.1 mV s^−1^. (Adapted from ref. 72, copyright 2021 Royal Society of Chemistry).

In Fig. 15 the potential difference *E*_diff_ peak value with the highest current value between the discharge and charge cycle, 4.2 V *vs.* Na^+^/Na, are represented with respect to the number of cycles. The value of *E*_diff_ can give understanding into the redox process reversibility. At more *E*_diff_ value, the more the redox procedure will be more irreversible.

**Fig. 16:**
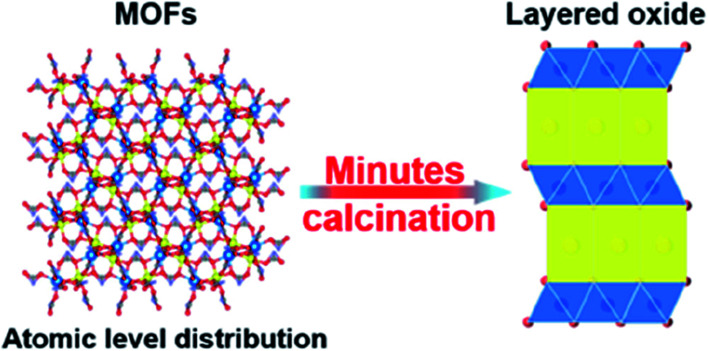
Schematic illustration of the preparation method of MOF based TMO cathode material. (Adapted from ref. 88, copyright 2022 American Chemical Society).

The lithium doped TMO was successfully synthesized by Yang's group. P2–Na_0.66_Li_0.18_Fe_0.12_Mn_0.7_O_2_ displayed high capacity of 190 mA h g^−1^ with ∼87% capacity retention over 80 cycles.^84^ The stability is ascribed to the reversible migration of lithium while cycling and reduction of P2–O2 transformation.^85,90^ The appropriate sodium content is important to achieve remarkable electrochemical performance. P2-type cathode-Na_0.85_Li_0.12_Ni_0.22_Mn_0.66_O_2_ containing high sodium content exhibited plateau-free and fast Na^+^ mobility with low volume expansion (∼1.7%). This material delivered 123.4 mA h g^−1^ capacity with outstanding rate capability and 85.4% capacity was retained over 500 cycles.^86,87^ Recently, the metal–organic frameworks (MOFs) based layered TMOs were developed which is the cost-effective synthesis method for high performance cathode materials as shown in Fig. 16. The homogeneous distribution of atoms in MOFs allows fast phase transition during calcination. This show outstanding electrochemical performance. Na_*x*_MnO_2_ synthesized using MOF precursor demonstrated the high specific capacity of 212 mA h g^−1^ with capacity retention of 84% over 100 cycles.^88^ Besides, P2–Na_0.7_Li_0.03_Mg_0.03_Ni_0.27_Mn_0.6_Ti_0.07_O_2_ exhibited high reversible specific capacity and excellent cyclic stability. This was due to the contribution of Ti, Mg and Li which provides high redox potential, structural stability, and smoothening of electrochemical curves respectively. Finally this was concluded that strategy of doping the elements is the rationale route to improve the electrochemical performance of the cathode material.^89^

### 4.3 O/P biphasic type transition metal oxides

**Fig. 17:**
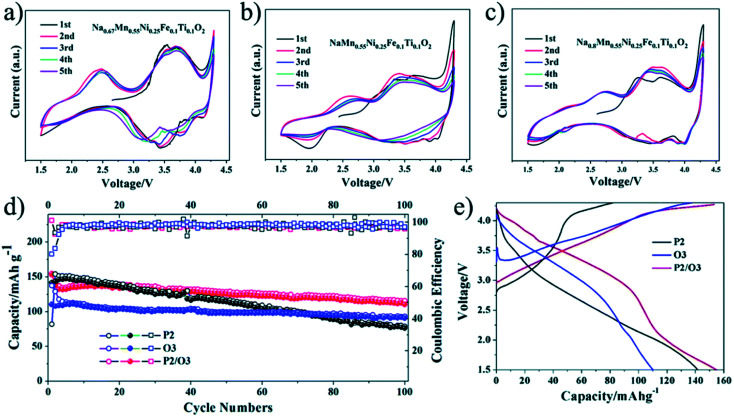
(a) CV plot of P2, (b) CV plot of O3, and (c) CV plot of O/P two phase sample; (d) capacity *vs.* cycle number plot of the samples and (e) voltage-first discharge capacity plots. (Adapted from ref. 90, copyright 2021 Royal Society of Chemistry).

The electrochemical performance of the O, the P, and the O/P-phase samples were analysed by Wang Kai *et al.* using CV as shown in Fig. 17(a) and (b). The CV plot of the P2-phase sample shows two well-specified oxide peaks at about 3.7 and 2.4 V, relating to Mn^3+/4+^ and Ni^2+/3+^ transition. Additionally, the space between the Mn^3+/4+^ redox peaks is moved to greater voltage than the P2 type sample related to a greater energy density of the O3 type sample. The biphasic sample shows greater shift of the reduction and oxidation peaks towards a greater voltage Fig. 17(c). Broad and smooth peaks after 1st cycle are a strong signal of a solid-state reaction and greater energy density of the O/P two phase materials than the single-phase P2 type material. The cycling characteristics of the materials is displayed in Fig. 17(d) and (e). The O/P two phase sample displays the finest cycling properties. Even though, the pure P2-phase sample has a greater first discharge capacity compared to the pure O3-phase sample, the lower cycling stability outcomes in inadequate retention of capacity. Primarily, a distinct reduction of the reversible capacity of the O/P two phase sample is identified.^90^

**Fig. 18:**
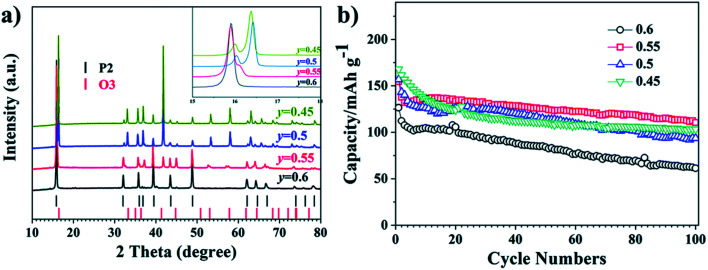
(a) XRD patterns of Na_0.8_Mn_*y*_Ni_0.8−*y*_Fe_0.1_Ti_0.1_O_2_ (*y* = 0.45, 0.5, 0.55, 0.6). (b) Cycling performance of Na_0.8_Mn_*y*_Ni_0.8−*y*_Fe_0.1_Ti_0.1_O_2_ (*y* = 0.45, 0.5, 0.55, 0.6). (Adapted from ref. 90, copyright 2021 Royal Society of Chemistry).

**Fig. 19:**
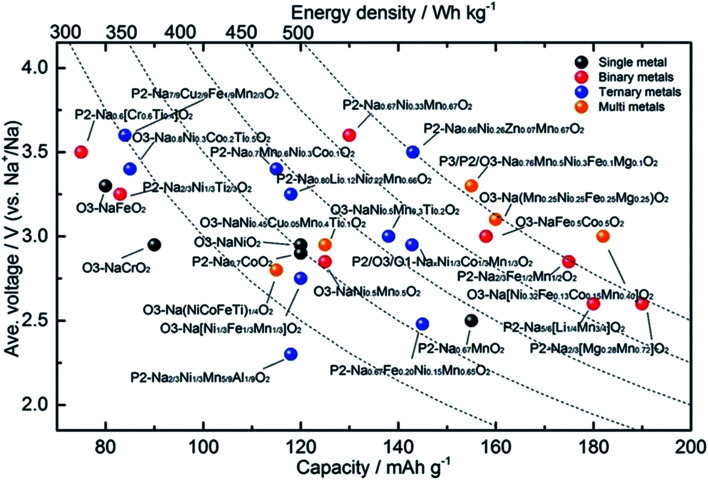
An electrochemical summary of various layered oxide sodium-ion cathode materials, comparing voltage, capacity and energy density. (Adapted from ref. 91, copyright 2020 Wiley Online Library).

Wang Kai *et al.* reported synthesis of intergrown O/P-type two-phase layered oxides as high-performance cathode for SIBs. Two phase Na_0.8_Mn_*y*_Ni_0.8−*y*_Fe_0.1_Ti_0.1_O_2_ (*y* = 0.45, 0.5, 0.55, 0.6) positive electrode materials are prepared by solid-state method. Electrochemical experiments of the prepared sample reveal improved characteristics of the O/P two phase materials in a SIBs than single O3 or P2 phases, confirming the advantageous outcome of the intergrowth of O3 and P2 materials. All O/P two phase composites Na_0.8_Mn_*y*_Ni_0.8−*y*_Fe_0.1_Ti_0.1_O_2_, (*y* = 0.45, 0.5, 0.55, 0.6), P2-type Na_0.67_Mn_0.55_Ni_0.25_Fe_0.1_Ti_0.1_O_2_, and O3-type NaMn_0.55_Ni_0.25_Fe_0.1_Ti_0.1_O_2_ materials were synthesized by a solid-state method. The Mn to Ni ratio of the Na_0.8_Mn_*y*_Ni_0.8−*y*_Fe_0.1_Ti_0.1_O_2_ was changed, with *y* = 0.45, 0.5, 0.55, 0.6. Fig. 18(a) displays the XRD pattern analysis of the various samples, verifying the existence of both O3 and P2 phases, collectively with the preferred two-phase composition, Na_0.8_Mn_0.55_Ni_0.25_Fe_0.1_Ti_0.1_O_2_. Disparity in the ratio of O3/P2 two phase materials will also affect the electrochemical characteristics. Fig. 18(b) displays the cyclic performances of the different samples. Even though three mixed O/P phases have better first discharge capacity than single-phase material, after 10 number of cycles the greatest retention of discharge capacity is seen for Na_0.8_Mn_0.55_Ni_0.25_Fe_0.1_Ti_0.1_O_2_. Therefore, the Na_0.8_Mn_0.55_Ni_0.25_Fe_0.1_Ti_0.1_O_2_ is most favourable two-phase material in this series.^90^ The diffraction pattern of the Na_0.67_Mn_0.55_Ni_0.25_Fe_0.1_Ti_0.1_O_2_ sample shows the existence of a pure P2 type phase, which fits nicely with the general P2 type structure for the synthesized sample. For O3-phase NaMn_0.55_Ni_0.25_Fe_0.1_Ti_0.1_O_2_, there are little peaks of impurity in the XRD pattern of material that might be the outcome of some impurities of TMOs or other layered type structures difficult to detect individually because of their low reaction concentrations. The XRD pattern of the two-phase material includes the peaks of both the O3 and P2 phases that fit to the space groups *R*3̄*m* and *P*6_3_/*mmc*. High crystallinity of the material is signified by the sharp peaks in the XRD pattern. The two-phase material gives higher capacity than the P2-phase material after 20 cycles owing to its improved cycling stability. After the 76th cycle, the pure O3-phase sample also shows a greater capacity compared to the P2-phase material. The O/P intergrown Na_0.8_Mn_0.55_Ni_0.25_Fe_0.1_Ti_0.1_O_2_ structure demonstrated a capacity retention of 80.2% keeping 110 mA h g^−1^, after 100 cycles that is much greater compared to the pure P2-phase sample (almost 53.8%). After 100 cycles, the first discharge capacity of the O3 phase sample is less (97 mA h g^−1^) owing to its less initial discharge capacity. The O/P two phase material showed the greatest energy density of 451 W h kg^−1^ owing to the higher voltage and reversible capacity. Furthermore, the O/P two phase material showed well rate performance than the O3- and P2-phase materials. Fig. 17(e) displays the first charge–discharge plots of the materials. The initial charge capacity of the P2-phase sample is 82 mA h g^−1^, that is far lesser compared to the subsequent discharge capacity of 142 mA h g^−1^. The first charge capacity of for the pure O3-phase material 138 mA h g^−1^ that is greater than the corresponding discharge capacity of 110 mA h g^−1^, that can be associated to its adequate sodium reserves. Expectedly, the O/P phase material provided the greatest initial discharge capacity of almost 153 mA h g^−1^. Furthermore, the charge–discharge plots display that O/P two phase Na_0.8_Mn_0.55_Ni_0.25_Fe_0.1_Ti_0.1_O_2_ demonstrated greater voltage of discharge with better energy density compared to the pure phases. A greater reversible capacity of 154.6 mA h g^−1^ with good initial coulombic efficiency of the two-phase material achieved with an outstanding retention in capacity (80.2%) at 0.1C after 100 cycles. Furthermore, the O/P biphasic material showed greater energy density and greater rate performance compared the pure O3 and P2 phases. The excellent electrochemical characteristic of the two-phase sample is ascribed to the harmonious properties between the intergrown O3 and P2 phases improving both ion mobility and stability.^82^ The plot of various TM*O*s are summarized in terms of capacity, energy density and average voltage in Fig. 19.

### 4.4 Other type transition metal oxides

Along with the layered TMOs, mono-layered, spinel and tunnel structured metal oxides also exhibit remarkable electrochemical performance. Even so, mono-layered TMOs belong to the superlattice ordering system.^92^

**Fig. 20:**
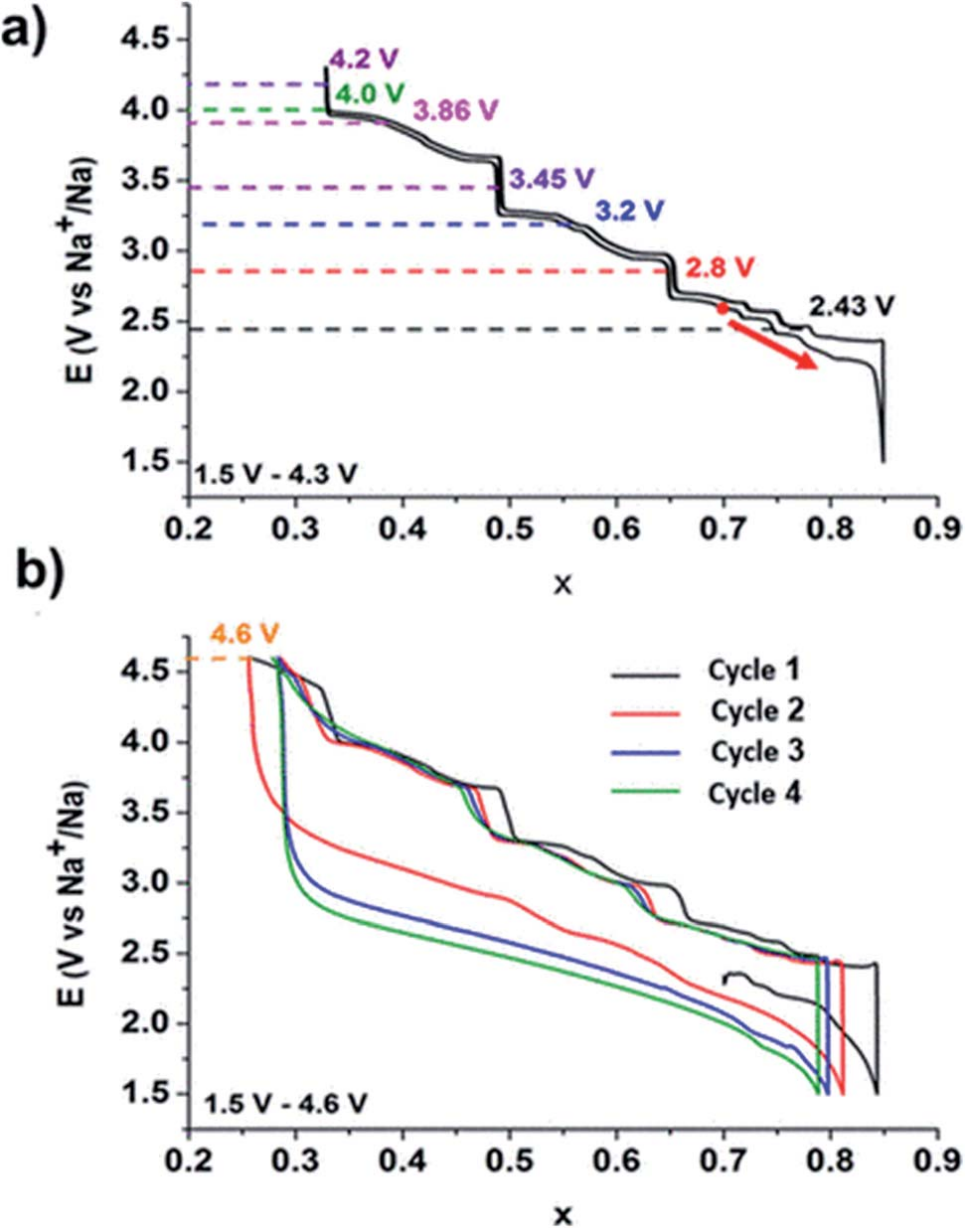
Galvanostatic cycling curve of a Na//P2–Na_*x*_CoO_2_ battery representing the phase diagram. (Adapted with permission from ref. 93, copyright 2022 American Chemical Society).

Fig. 20 represents the charge/discharge profiles displayed by these materials which shows multiple voltage plateaus that associated with the cycle performance of the cathode materials. Solid solution TMOs shows that lattice parameters vary linearly with the Na content. However, they exhibit excellent electrochemical performance due to presence of the P-type and O-type structures. Recently, these materials are proposed for SIBs. Monoclinic NaMnO_2_ displayed good electrochemical properties determined with computational method as well as experimentally.^94^ Noha Sabi *et al.* synthesized Ti substituted NaCoO_2_ and delivered initial charge capacity of 108 mA h g^−1^ with remarkable cyclic stability. This study gave insights about the sodiation/desodiation mechanism of the this compounds showed the effect of inactive doping in the oxide material.^95^ Some studies reported that the mono-layers of the transition metal carbides/nitrides (MXenes) enhances the accessibility to the surface.^96^ The study has revealed that MXenes exhibit excellent electrochemical performance due to its exceptional properties such as electrical conductivity, thermal stability and mechanical strength.^97^

**Fig. 21:**
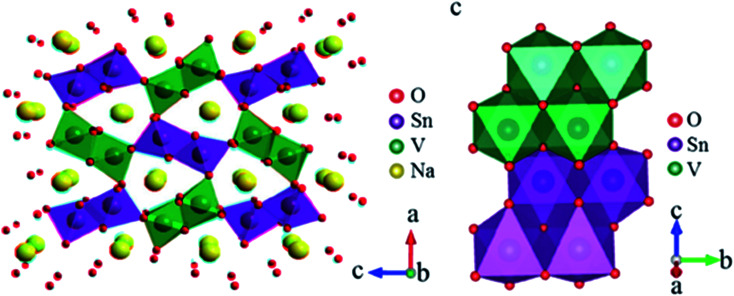
Crystal structure of post-spinel NaVSnO_4_. (Adapted with the permission from ref. 100, copyright 2017 Wiley Online Library).

High operating voltage, thermal stability and good specific capacity make spinel-like LiMn_2_O_4_ a promising cathode material for LIBs. But, analogous spinel-like NaMn_2_O_4_ is challenging as it is thermally instable. They transform spinel to post-spinel structure at high pressure offering new pathway for application in rechargeable battery cathodes.^98^ NaVSnO_4_ post-spinel crystal structure composed of VO_6_ and SnO_6_ octahedrons of metal oxides as shown in Fig. 21.^99^

**Fig. 22:**
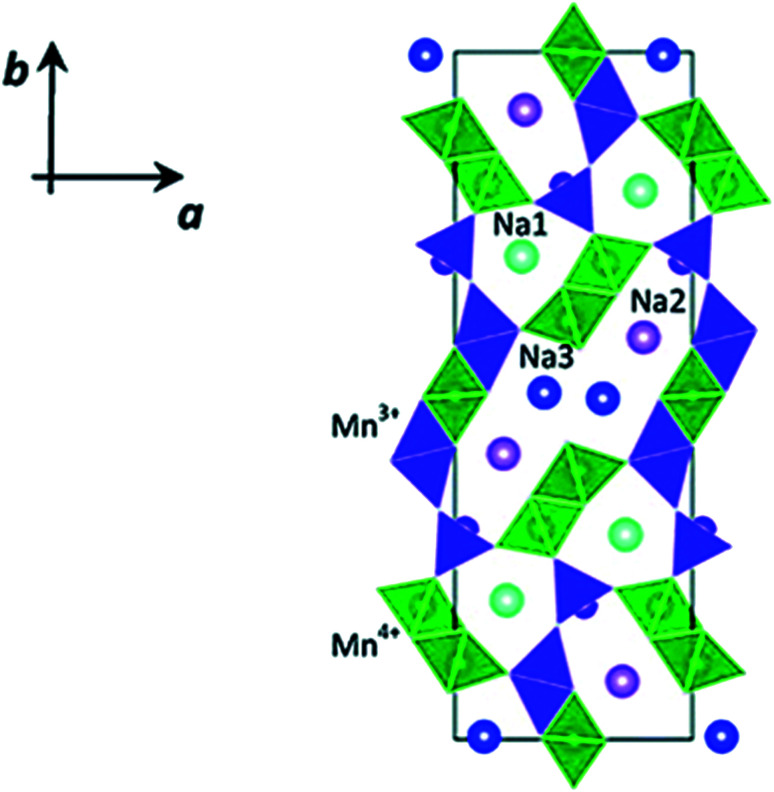
Crystal structure of tunnel-type Na_0.44_ MnO_2_. (Adapted with the permission from ref. 103, copyright 2022 Royal Society of Chemistry).

Apart from these crystal structures, tunnel-type TMOs (Fig. 22) have been extensively studied for SIB application. The Na_*x*_MnO_2_ tunnel oxide attracted much attention as Na^+^ insertion host for Mn based TMOs with less Na content. It offers higher structural stability as well as Na^+^ diffusion rate compared to layered TMO Na_*x*_MnO_2_.^101^ But, the intercalation potential of these material is low. This leads to the substitution of element at transition metal site as effective way to get desirable properties of the cathode compounds.^102^

The single-phase material frequently exhibits constrained and imbalanced electrochemical properties, even though layered materials are promising cathodes for rechargeable SIBs. For instance, the P2 structure's wide, triangularly shaped Na-ion transport channel exhibits low migration energy barrier and good rate performance, but the structure's unfavourable Na/vacancy ordering and P2–O2 phase transition result in poor structural stability and worse capacity retention.^104^ The O3 structure has various drawbacks, including low air stability, difficult phase transitions, and a high energy barrier for Na diffusion. Nevertheless, it is a sufficient Na-ion storage that can feed enough Na ions to a full cell. The tunnel structure has favourable kinetic dynamics and strong structural stability, however major improvement is required for its small capacity. To concentrate advantages and overcome disadvantages of the single-phase material to attain good detailed electrochemical characteristics, composite-structure materials (CSMs) that mix two or more crystal structures have garnered considerable interest. The two types of CSMs under investigation are layered-tunnel CSMs and layered–layered CSMs.^105,106^

Some of the existing and upcoming challenges related to layered SIBs are connected to the development of high performing cathode materials. Still, the added possibility afforded by the two different environments of sodium inside the O3|P2 layered materials consequence in extra complexities of structures upon cycling of battery. A basic and comprehensive understanding of this structural development over thousands of cycles is essential for the improvement of cycling, increased lifetime performance and to mitigate degradation. Notable variations in performance can result from small replacements and some of them are reviewed in Table 3. P2-based systems can meet the best power performance due to the specific sodium-ion surroundings that permit facile ionic mobility over rectangle-shaped faces between neighbouring trigonal prismatic environments. In LIBs, this diffusion mechanism is unavailable, indicates that the improvement of P2-based SIBs may provide a higher coefficient of diffusion and, accordingly higher rate of discharge and charge than for LIBs.^56^ There are possibilities to manufacture sustainable green batteries by layered O3|P2 type cathodes made from earth-abundant materials. Table 3 shows the development of TMO (NaMO_2_) cathode material for SIBs with different types of doping. In this review article, cycle number, voltage range, current density, first discharge capacity and capacity retention of the different phases of materials have been compared. It can be seen from Table 3 that even a small fraction of doping in stoichiometric ratio can change the properties of materials.

**Table 3:** Development of cathode for SIB

Composition	Type	Voltage (V)	First discharge capacity [mA h g^−1^]	Capacity retention (%), cycle number	References
Na_0.6_Li_0.2_Mn_0.8_O_2_	P2	2–4.6	190 at 1C	100, 100	107
Na_0.45_Ni_0.22_Co_0.11_Mn_0.66_O_2_	P2	1.5–4.6	200 at 0.1C	80, 100	108
Na_0.55_Ni_0.1_Fe_0.1_Mn_0.8_O_2_	P2	1.5–4.3	221.5 at 0.1C	75, 100	109
Na_0.67_Mn_0.65_Ni_0.2_Mg_0.15_O_2_	P2	2–4.3	125 at 0.1C	94, 100	110
Na_2/3_Co_2/3_Mn_2/9_Ni_1/9_O_2_	P2	2–4.5	140 at 0.05C	85, 10	83
Na_2/3_Ni_1/6_Mn_2/3_Cu_1/9_Mg_1/18_O_2_	P2	2.5–4.15	64 at 0.1C	81.4, 500	111
Na[Li_0.05_(Ni_0.25_Fe_0.25_Mn_0.5_)_0.95_]O_2_	O3	1.75–4.4	180 at 0.1C	90, 20	112
Na_0.9_Ca_0.05_Ni_1/3_Fe_1/3_Mn_1/3_O_2_	O3	2–4	127 at 0.1C	92, 200	113
Na_0.75_Ni_0.82_Co_0.12_Mn_0.06_O_2_	O3	2–4	171 at 0.1C	65, 400	114
Na_4_FeRuO_6_	O3	2–4	120 at 0.2C	80, 100	115
Na_0.67_Fe_0.425_Mn_0.425_Mg_0.15_O_2_	P2/O3	1.5–4.2	98 at 0.1C	95, 50	116
Na_2/3_Li_0.18_Mn_0.8_Fe_0.2_O_2_	P2/O3	1.5–4.2	125 at 0.1C	70, 100	117
Na_0.7_Li_0.06_Mg_0.06_Ni_0.22_Mn_0.67_O_2_	P2/P3	2–4.4	129 at 0.2C	97, 50	118
Na_0.66_Mn_0.5_Co_0.5_O_2_	P2/P3	1.5–4.3	156 at 1C	55, 100	119
Na_0.6_Mn_0.75_Ni_0.25_O_2_	P2/P3	2–4.1	101 at 0.2C	68, 500	120
Na_0.66_Mn_0.9_Mg_0.1_O_2_	P2	2–4.5	162.9 at 1C	65, 100	121
Na_0.8_Co_0.4_Ti_0.6_O_2_	O3	1.1–4	100 at 0.1C	80, 100	122
Na_2/3_[Fe_0.22_Mn_0.78_]O_2_	P2	1.5–4.3	187 at 0.1C	91, 100	123
NaNi_0.5_Mn_0.5_O_2_	O3	2.2–3.8	125 4.8 mA g^−1^	75, 50	124
Na_2/3_Mn_0.8_Fe_0.1_Ti_0.1_O_2_	P2	2–4	144.16 at 0.1C	87.7, 300	125
Na_0.67_Cu_0.28_Mn_0.72_O_2_	P2	2–4.5	109 at 0.1C	98, 50	126
Na_1.2_Mn_0.4_Ir_0.4_O_2_	O3	1.5–4.4	133 at 0.1C	60, 50	127
Na_0.67_Al_0.1_Mn_0.9_O_2_	P2	2–4	175 12 mA g^−1^	86, 100	128
Na_2/3_[Mn_0.8_Ni_0.2_]O_2_	P2	2–4.3	162 at 0.1C	75, 100	129
Na_0.6_Ni_0.22_Al_0.11_Mn_0.66_O_2_	P2	1.5–4.6	252 20 mA g^−1^	80, 50	130
NaNi_1/3_Fe_1/3_Mn_1/3_O_2_	O3	2–4	136 at 0.1C	80, 100	131
Na_0.78_Al_0.05_Ni_0.33_Mn_0.60_O_2_	P2	2–4.5	131.9 at 0.1C	83.9, 50	132
Na_0.67_Fe_0.20_Ni_0.15_Mn_0.65_O_2_	P2	2–3.8	145 at 0.1C	55, 900	133
NaNiO_2_	O3	1.25–3.75	123 at 0.1C	93.5, 20	134
Na_0.67_[Ni_0.1_Fe_0.1_Mn_0.8_]O_2_	P2	1.5–4.3	220 at 0.1C	80, 200	135
Na_0.97_Cr_0.97_Ti_0.03_O_2_	O3	2–3.6	125 at 0.2C	96, 100	136
Na_0.86_Co_0.475_Mn_0.475_Ti_0.05_O_2_	P2	1.5–4	111.8 at 0.5C	81.4, 200	137
Na_0.82_Mn_1/3_Fe_2/3_O_2_	O3	1.5–3.8	132 at 0.02C	92, 12	138
Na_0.66_Li_0.18_Mn_0.71_Mg_0.21_Co_0.08_O_2_	P2	1.5–4.5	166 20 mA g^−1^	82, 100	139
NaCu_0.22_Fe_0.30_Mn_0.43_Ti_0.05_O_2_	O3	2.5–4.05	90 at 0.2C	96, 200	140
Na_2/3_MnO_2_	P2	2–4.3	164 at 0.1C	50, 50	141
NaCu_0.22_Fe_0.30_Mn_0.48_O_2_	O3	2.5–4.1	85 at 0.2C	84, 200	142
Na_2/3_[Mn_0.8_Co_0.2_]O_2_	P2	1.5–4.6	175 at 0.1C	90, 300	143
NaCu_1/9_Ni_2/9_Fe_1/3_Mn_1/3_O_2_	O3	2–4	127 10 mA g^−1^	88, 100	144
Na_0.67_Ni_0.2_Fe_0.15_Mn_0.65_O_2_	P2	1.5–4.3	207 at 0.1C	31, 50	145
Na_0.83_Cr_1/3_Fe_1/3_Mn_1/6_Ti_1/6_O_2_	O3	1.5–4.1	161.4 at 0.1C	35, 100	146
Na_0.66_Co_0.22_Mn_0.44_Ti_0.34_O_2_	P2	1.5–4.3	135 at 0.1C	53, 200	147
NaCr_1/3_Fe_1/3_Mn_1/3_O_2_	O3	2–4.1	165 at 0.03C	64, 10	148
Na_0.55_[Ni_0.1_Fe_0.1_Mn_0.8_]O_2_	P2	1.5–4.3	221.5 12 mA g^−1^	75, 100	109
NaMn_0.48_Ni_0.2_Fe_0.3_Mg_0.02_O_2_	O3	1.5–4.2	160 at 0.05C	99, 100	149
Na_0.66_[Ni_0.13_Mn_0.54_Co_0.13_]O_2_	P2	2–4.7	120 at 1C	90, 150	150
Na[NiCoMnTi]_1/4_O_2_	O3	2–3.9	116 at 0.1C	75, 400	151
NaNi_0.45_Cu_0.05_Mn_0.4_Ti_0.1_O_2_	O3	2–4.0	124 at 0.1C	70.2, 500	62
Na_0.44_Mn_0.6_Ni_0.3_Cu_0.1_O_2_	P2	1.5–4	149 at 0.1C	80, 50	152
[Na_0.67_Li_0.2_][Fe_0.4_Mn_0.4_]O_1.6_	O3	1.5–4.5	162 10 mA g^−1^	72, 200	153
Na_0.67_Ni_0.233_Mn_0.67_Mg_0.10_O_2_	P2	2–4.5	120 at 0.1C	81.7, 100	154
Na[Li_0.05_Mn_0.5_Ni_0.30_Cu_0.1_Mg_0.05_]O_2_	O3	2–4	172 at 0.1C	70.4, 1000	155
Na_2/3_Ni_1/3_ Mn_5/9_Al_1/9_O_2_	P2	1.6–4	116.7 at 0.1C	77.5, 100	156
NaNi_0.45_Mn_0.2_Ti_0.3_Zr_0.05_O_2_	O3	2–4	141.4 at 0.05C	70, 200	68
Na_2/3_Ni_1/3_Mn_2/3_O_2_	P2	1.5–4	166.7 at 0.1C	81, 500	157
NaNi_0.5_Mn_0.2_Ti_0.3_O_2_	O3	2–4	135 at 0.05C	85, 200	124
Na_0.85_Li_0.10_Ni_0.175_Mn_0.525_Fe_0.2_O_2_	O3	2–4.5	157 at 0.1C	88, 100	158
Na[Li_0.05_(Ni_0.25_Fe_0.25_Mn_0.5_)_0.95_]O_2_	O3	1.75–4.4	180 at 0.1C	92.1, 20	112
Na_0.72_Li_0.24_Mn_0.76_O_2_	P2	1.5–4.5	231 at 0.05C	40.7, 80	159
Na_0.9_Ca_0.05_Ni_1/3_Fe_1/3_Mn_1/3_O_2_	O3	2–4	126.9 at 0.1C	91.8, 200	113
Na_0.75_Ni_0.82_Co_0.12_Mn_0.06_O_2_	O3	2–4	171 at 0.1C	65, 400	114
Na_0.67_Mn_0.8_Ni_0.1_Mg_0.1_O_2_	P2	1.5–4.2	171 at 0.05C	79, 50	75
NaFe_0.55_Mn_0.44_Nb_0.01_O_2_	O3	2–4	127 at 0.1C	65.6, 200	160
NaLi_0.1_Ni_0.35_Mn_0.55_O_2_	O3	2–4.2	128 12 mA g^−1^	85, 100	161
NaNi_0.5_Mn_0.5_O_2_	O3	2–4	141 at 0.05C	90, 100	162
Na_2/3_MnO_2_	P2	2–3.8	155 at 1C	86.5, 225	133

**Fig. 23:**
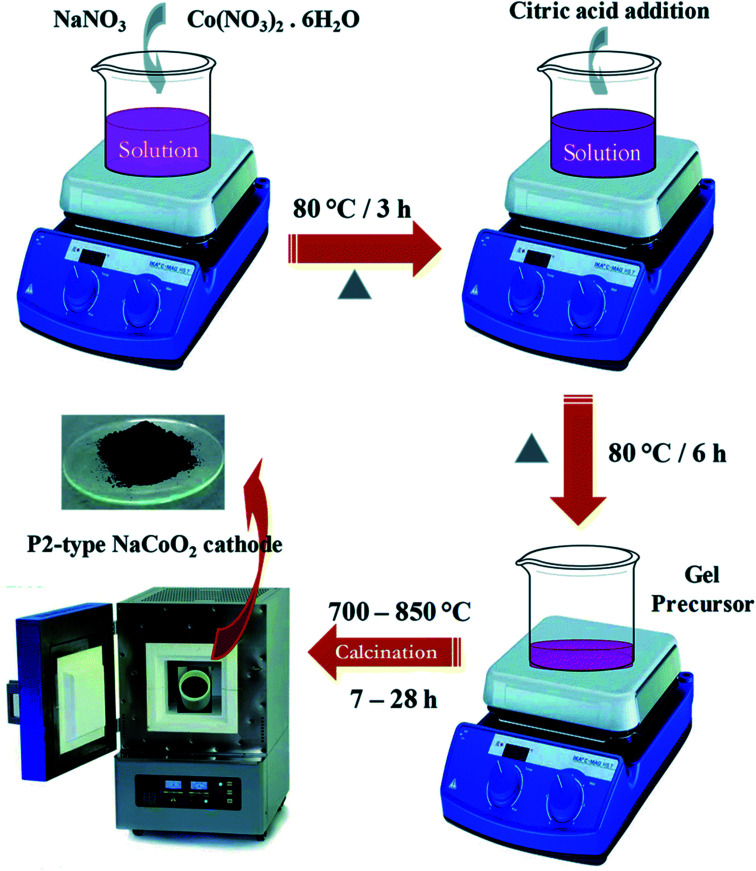
Synthesis scheme of the NaCoO_2_ cathode. (Adapted from ref. 163, copyright 2021 Royal Society of Chemistry).

Some important processes used to synthesize material for battery are, solid-state method, novel sol–gel method, reverse microemulsion method, sol–gel method, combustion method, electro-spinning method, hydrothermal method, combustion method, oxalate precursor-based method, combustion method. Table 4 shows the change in the electrochemical performance of Na_0.44_MnO_2_ made by various processes as cathodes for SIBs. Properties of materials made from different processes will vary as synthesis conditions are different in all operations. For example, synthesis process of the NaCoO_2_ cathode is shown in Fig. 23. In this process obtained gel precursor was dried at 120 °C for 24 h and calcined at various temperatures (700–850 °C), yielding the product of the P2-type NaCoO_2_ material.^163^

**Table 4:** Summary of Na_0.44_MnO_2_ made by various processes as cathodes for sodium-ion batteries

Morphology	Synthetic method	Voltage (V)	Cycle	Specific capacity (mA h g^−1^)	References
Slab	Solid-state method	2.0–3.8	40	100 at 0.2C	164
Nanoplate	Novel sol–gel method	2.0–4.0	100	105 at 0.5C	101
Rod	Reverse micro-emulsion method	2.0–3.8	100	97.6 at 0.1C	165
Nanorod	Sol–gel method	2.0–4.0	200	75 at 0.2C	166
Rod	Combustion method	2.0–4.0	100	105 at 1C	167
Nanofiber	Electro-spinning method	1.5–4.0	140	80 at 0.42C	168
Nanowire	Hydrothermal method	2.0–4.0	20	90 at 8.3C	169
Rod and slab	Combustion method	2.0–4.0	300	100 at 4C	170
Rod	Oxalate precursor-based method	2.0–4.2	100	112 at 0.5C	171
Stick	Combustion method	2.0–4.0	100	88 at 1C	172

## 5. Current challenges and future directions

The important challenges for the TMO are the structural instability (caused by humidity sensitivity), irreversible phase transitions, and electrochemical degradations. And volume transformation during the redox reaction. This holds up the use of TMOs. By using *in situ* XRD, one can see that P2-type materials often go through a P2–O2 phase transition while O3-type materials go through a more complicated phase transition. The occurrence of this irreversible phase shift will cause the cathode material's structural degradation and capacity loss. Additionally, because the laminate materials are so sensitive to their surroundings, moisture and airborne CO_2_ will have some effect on them. The materials' electrochemical characteristics will degrade as a result of side reactions with the electrolyte. The current and future challenges the SIBs faces are mainly related to the complexity of cathode materials which are relatively underperforming in comparison with LIBs in terms of energy density, capacity, power density and cyclability. The alternative way to find suitable element addition to NaCoO_2_ which is redox inactive however boost the efficiency of SIBs. Cobalt is another expensive and towards toxic side hence the best alternative to replace Co with Ni, Mn, and Ti to make stability and enhance the performance of the SIBs, many reports already noted about this. This will surely reduce the manufacturing cost. However, the phase controlling of P2 and O3 in this layered structure is challenging which directly affect the performance of the SIBs. Another challenges that the SIBs are comprising with the anode materials which affecting the performance. The hard carbon will be best option to enhance the performance however the suitable and economical technique to grow hard carbon should be developed.

### 5.1 Irreversible phase transformation

**Fig. 24:**
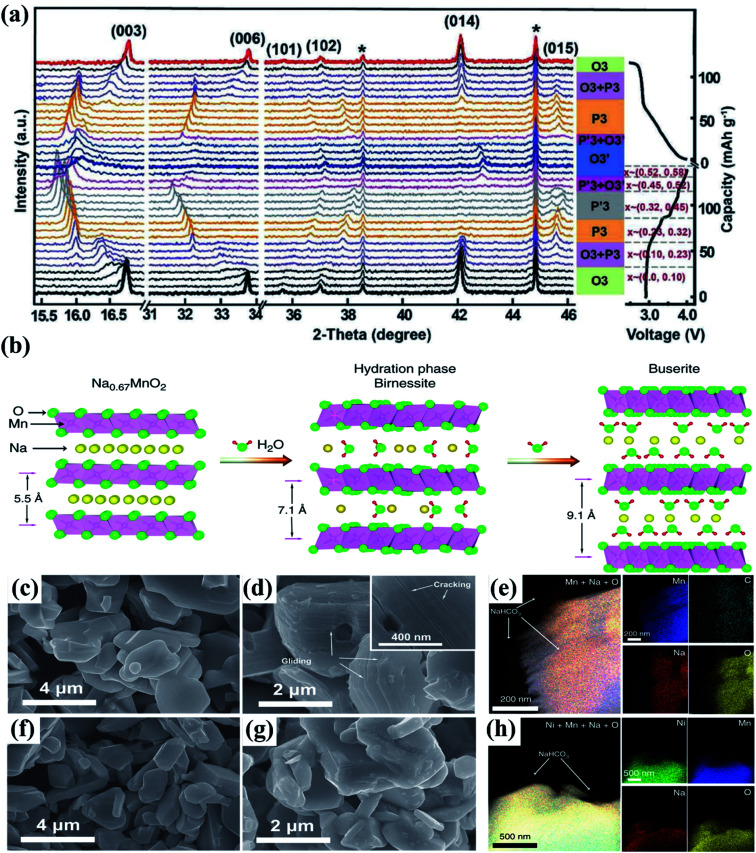
(a) *In situ* XRD pattern of NaFe_0.45_Co_0.5_Mg_0.05_O_2_ (NaFCM) during the first cycle of charging and discharging at 2.0–4.5 V. (Adapted from ref. 174, copyright 2017 Wiley-VCH). (b) Influence of water absorption on the structure of the P2–Na_0.67_MnO_2_ material; the SEM images of (c) pristine and (d) relative humidity (RH) 93% + CO_2_ exposed Na_0.67_MnO_2_ powder, (e) the EDS mapping results of exposed Na_0.67_MnO_2_, indicating that NaHCO_3_ is formed on the particles' surface. SEM images of (f) pristine and (g) RH 93% + CO_2_ exposed Na_0.67_Ni_0.33_Mn_0.67_O_2_ powder, (h) the EDS mapping results of RH 93% + CO_2_ exposed Na_0.67_Ni_0.33_Mn_0.67_O_2_ samples, NaHCO_3_ particles are also observed on the surface of exposed Na_0.67_Ni_0.33_Mn_0.67_O_2_. (Adapted from ref. 182, copyright 2020, all rights reserved).

In general, during the electrochemical cycle, the O3 and P2 phases go through a number of phase transitions affecting multiple stacking arrangements of the oxygen layer.^173^ The P2 phase often transitions to the O2 phase throughout the charging and discharging process as a result of the sliding of TMO_6_ octahedral layers with the removal of Na^+^. These issues cause the crystal structure to significantly shrink and the space between the layers to decrease. In contrast, Na^+^ initially settles in the shared TMO_6_ octahedral position with the edges during the O3 phase. The Na^+^ in the prism's core becomes energy stable when the sodium ions are partially removed from the O3 phase. This is related to the development of vacancies, which is analogous to the P2 phase. The TMO_2_ sheet is then moved while maintaining the TM–O bond, creating a wide prism centre. As a result, the usual oxygen build-up shifts from “ABCABC” to “ABBCCA,” which is a P3 phase. For NaNi_0.5_Mn_0.5_O_2_, the P2 phase typically exhibits a P2–O2 phase transition that can be seen by *in situ* XRD, however the O3 phase frequently experiences a more complex phase transition as shown in Fig. 24a than the P2 structure,^174,175^ such as O3 → O′3 → P3–P3′′.^176–180^ Therefore, P2 structured oxides always exhibit strong cycle stability and rate capacity because of their good structural integrity and low diffusion barrier.^181^

### 5.2 Structural instability due to humidity sensitivity

After being exposed to air, the layered cathode materials' hygroscopic qualities cause poor battery performance and higher transportation costs. Oxides with multilayer structures are extremely susceptible to environments with moisture. For instance, when its humid outside, water molecules will infiltrate the sodium layer, dramatically altering its structural makeup. CO_2_ will also have some effect on these materials because it can react with the oxides to produce electrochemically inert NaOH and Na_2_CO_3_. A thorough and organised understanding of the reaction mechanism of layered structure oxides in moist air is still lacking.^183^ As research objects in 2020, Zou *et al.*^182^ used Na_0.67_Ni_0.33_Mn_0.67_O_2_ and Na_0.67_MnO_2_ materials to examine structural evolution in a moisture environment as shown in Fig. 24(b). The chemical and structural changes that occur when materials are exposed to ambient air were carefully investigated using a variety of spectroscopy and electron microscopy characterization techniques, including *in situ* XRD, solid-state NMR, TOF-SIMS, and XRD as depicted in Fig. 24(c)–(h). This study is the first to suggest and demonstrate that the first cycle charging reaction potential can be utilised as the judging criteria for layered oxides' air stability, which will speed up the creation of high-performance cathodes for SIBs.

### 5.3 Electrochemical degradation

The layered oxide cathode experiences an irreversible phase change and water erosion, which causes rapid capacity decay and restricts the reversible capacity. Due to the following factors, the electrochemical performance of layered structure cathodes is still insufficient for large-scale device applications. First, the active components may be lost because of the transition metal ions dissolving into the electrolyte. The presence of Mn^3+^ in layered manganese-based cathode materials will cause the Mn–O bond to extend in a certain direction. Severe structural deformation results from the crystal structure's asymmetry. After Na^+^ is removed from the solid matrix, vacancies are created, creating areas for H_2_O/H insertion to create a protonated phase. The sodium ion diffusion coefficient may be significantly decreased because of this occurrence blocking ion transport routes. For instance, the capacity retention from the second cycle is as high as 87.7% for the P2-phase Na_2/3_Mn_0.8_Fe_0.1_Ti_0.1_O_2_ pristine electrode, whereas the moisture-exposed electrode only achieves 70% capacity retention.^184–186^

Researchers have been working diligently to find electrode materials with good performance to address the major challenges outlined before. Synthesis method, elemental doping, structural modulation, surface modification, and composite phase modulation are the key modification methodologies discussed here. Elemental doping is frequently employed to prevent irreversible phase changes during material cycling, surface coating can lessen electrolyte-particle side reactions, and the cathode material can be shielded from moisture and atmospheric CO_2_. In order to create satisfactory electrode materials, we can simultaneously design the structure and phase of the materials.

## 6. Techniques for improving the electrochemical performance of layered oxide materials

### 6.1 Synthesis methods to control and improve the morphology

The morphology and crystal structure of the layered oxide cathode material for SIBs influence the electrochemical performance of the battery. Thus, to preserve multiple phase formation, stable structure, low moisture sensitivity, high-level electronic conductivity, the changes of the structures is required. Generally, different synthesis methods for upgrading the crystal structure are developing to be a practical approach for lowering the sodium ion diffusion pathway and raising the surface area for Na^+^ ion intercalation/deintercalation. In general, the layered oxide cathode materials can be synthesized using the solid-state method. But the materials achieved by this method have not given good electrochemical performance. Therefore, the simple and most familiar synthesis process used is sol–gel method. The electrochemical performance is better as compared to other synthesis method, but this technique consumes most of the day which limits its useful applications. For making a small quantity of material and to obtain the ideal nanostructure, the hydrothermal method is also beneficial, however it does not give good electrochemical properties. Fu *et al*. synthesized Na_0.44_MnO_2_ nanorods with different solvents a raw material: sodium acetate (CH_3_COONa), acetic acid solution (CH_3_COOH), aqueous solution of manganous nitrate (Mn(NO_3_)_2_) by sol–gel method^166^ which show better electrochemical performance as compared to other methods. Every method has its own comparative advantages, has various conditions for cost and manufacture situation, as well as has some effects on the crystallinity, particle size, morphology, and phase purity of materials. Table 5 gives a concise difference of the merits and demerits of frequently used synthesis methods. Hence, to improve well layered TMO materials, the synthesis process requires to be constantly enhanced.^187,188^

**Table 5:** Merits and demerits of various synthetic processes for creating sodium ion-layered transition metal oxides

Synthetic method	Merits	Demerits
Sol–gel method	Low cost of production; good uniformity	Reaction times are too slow, difficult to globalise
Solid-state method	Simple operation, easy preparation	Materials are not uniform, difficult to manage the morphology and particle size
Combustion method	Fast and affordable synthesis method	Poor control of combustion process and morphology
Coprecipitation	Size and shape can be controlled more easily, and homogeneity is significantly higher	High synthesis costs, restrictive synthesis requirements
Spray drying	Fast drying times, easy management, and suitability for large-scale manufacture	The requirements for spray dryer are high

### 6.2 Doping

Doping of elements is one of the fundamental alteration strategies among the advancement studies on SIB cathode. Doping variation, also called replacement variation, is mainly to substitute portion of the equivalent site of the novel material structure by a minor number of additional ions or group structures and improving the performance and structure by creating vacancies, substantial voids, or change the lattice parameters. Corresponding to unique states of doping ions that can be divided into two ways: (1) cation doping and (2) anion doping. All has been showing effective for several cathodes in improving working voltage, enhancing rate capability, and increasing cycling stability.^189^

Generally, cation doping is commonly used to both oxide and polyanions, even though anion doping is generally found in polyanions. Additionally, doping of cations in a few cases is effective sufficient to achieve probable outcomes, although doping with only anions is observed to affect few side effects.^190^

#### Cation doping and anion doping

In TMOs, cation doping is mostly used to partially substitute sodium ions or other ion types with other metal cations. This strategy can accomplish numerous goals by picking the right replacement. To increase the cathode material's cycle performance, it first stabilizes the framework structure by preventing irreversible phase transitions.^176^

Second, active doping increases operating voltage or improves specific capacity by inducing reversible oxygen redox activity or by introducing new transition metal-based redox processes, which enhances the cathode material's energy density.^191^ Additionally, a smoother pathway and a faster Na^+^ diffusion coefficient are provided by more Na^+^ vacancies by ions with a higher valence state (such as Ti^4+^), improving the rate performance of the cathode material. This is made possible by doping the Na site with larger ions or shortening the length of the TM–O bond.^192,193^

TMOs differ from polyanion cathode materials in the mechanism and outcome of anion doping. Some elements, whose radius is comparable to that of oxygen, such F, S, Cl, *etc.*, are used to replace a minor quantity of O in TMOs. As a reference, Chen *et al.*^194^ suggested that F doping in Na_0.6_Mn_0.95_Ni_0.05_O_2_ can significantly enhance the activity of TM Ni_2_ by the creation of NiF bonds, increasing its working voltage and reversible specific capacity. Na_0.6_Mn_0.95_Ni_0.05_O_1.95_F_0.05_'s formation of the Na–F bond between sodium and fluorine stabilised the structure, preventing it from collapsing after Na deintercalation. As a result, cycle stability was increased, and after 960 cycles at 2C, the capacity retention rate was 75.0 percent. Even at 50 °C, the rate performance of the F-doped Na_0.6_Mn_0.95_Ni_0.05_O_2_ samples is superior to that of the pristine samples, particularly Na_0.6_Mn_0.95_Ni_0.05_O_1.95_F_0.05_.

### 6.3 Surface coating

During the charging, discharging, and holding of a cell, it has been discovered that the surface of various cathodes materials would interact with electrolyte or air. An organic-inorganic mixed insulating layer, also known as the cathode-electrolyte interface (CEI), consisting primarily of Na_2_CO_3_, would develop from such a reaction on the cathode. Since it consumes a lot of Na, the electronic conductivity decreased, and the electrochemical property was compromised.^195^ Many researchers suggested applying coating modifications to the surface of cathode materials, such as carbon coating,^196^ polymer coating,^197^ and inorganic compound coating^198^ to solve these issues. The cathode material can avoid reacting with the air and electrolyte by having a coating layer on its surface that can stop Na from escaping. Also, to improve the cathode material's electronic conductivity and improve its electrochemical capabilities, the coating layer is preferably made of a conductive material with good electrical conductivity.^199^ In addition to monolayer coating, double-layer coating has also been reported, although not in SIB cathodes, hence it is not covered in detail here.

### 6.4 Structure design and morphology

**Fig. 25:**
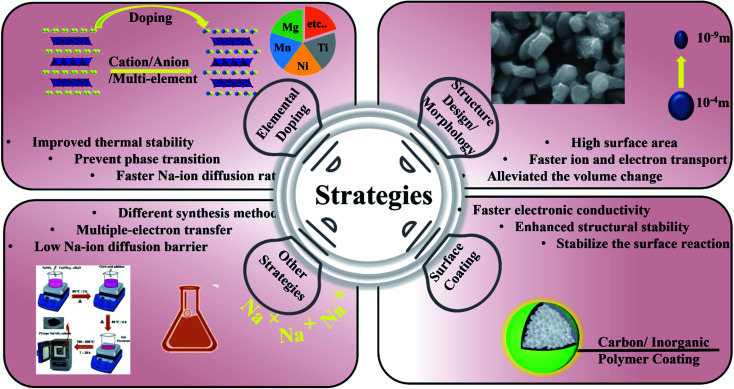
Strategies of elemental doping, structure design/morphology, surface coating and other processes to enhance the SIB performance.

Materials' morphology and structure are two important aspects that significantly affect their electrochemical characteristics. As a result, by carefully modifying the materials' internal structure and surface morphology, the electrochemical properties of cathode materials for SIBs can be maximized as summarized in Fig. 25.^200^ There are numerous studies on sodium TMOs that aim to increase the reaction area between electrolyte and electrode materials to speed up the transport of Na while maintaining a stable CEI. Strategies include creating porous architectures or manufacturing nanoscale electrode materials, both of which have helped sodium TMOs function more electrochemically.^201^

## 7. Conclusions

This review article provided the information about the development of LIB and SIB with drawback and advantages. It also deals with the complications exists with SIBs, including electrodes fabrication, assembling, economy factors. The cathode and anode material, synthesis, challenges, and alternatives are discussed in the review. The future prospects are also discussed, hopefully this review article will provide best insight of SIBs research community for the beginners and also for established researchers and industries too. In many battery technologies, layered O3|P2 cathode chemistry is at the leading position for the substantial improvement of the SIB performance over the past decades. The close structural similarities between layered lithium-ion cathodes and O3|P2 materials have assisted in modifying their commercial and scientific evolution. However, the higher complexity in the structure of O3|P2 materials, emerging from the capability of sodium ions to follow elongated octahedral and trigonal prismatic coordination, generates many difficulties and gives significant chances. The difficulties resulting from the combinational complexity of the permutation cations can also be possibilities to produce SIBs that can compete with the best LIBs. The opportunities are to develop SIBs that are reliable, economic, and competitive in performance with the best LIBs.

## Author contribution

Archana Kanwade: conceptualization, formal analysis, writing-original draft. Sheetal Gupta: formal research, writing-original draft and editing. Akash Kankane: writing and correcting the draft; Subhash Chand Yadav: formal analysis, writing-original draft. Manish Kumar Tiwari: formal analysis, writing-original draft. Abhishek Srivastava: formal analysis, writing-original draft. Jena Akash Kumar Satrughna: formal analysis, writing-original draft. Parasharam M. Shirage: designing the review, conceptualization, formal analysis, funding acquisition, supervision, writing-review, corresponding and editing.

## Conflicts of interest

There are no conflicts to declare.

## Supplementary Material

## References

[cit1] Sinha L., Shirage P. M. (2019). J. Electrochem. Soc..

[cit2] Palanisamy M., Boddu V. R. R., Shirage P. M., Pol V. G. (2021). ACS Appl. Mater. Interfaces.

[cit3] Boddu V. R. R., Puthusseri D., Shirage P. M., Mathur P., Pol V. G. (2021). Ionics.

[cit4] Yadav S. C., Sharma A., Devan R. S., Shirage P. M. (2022). Opt. Mater..

[cit5] Yadav S. C., Srivastava A., Manjunath V., Kanwade A., Devan R. S., Shirage P. M. (2022). Mater. Today Phys..

[cit6] Zhao Y., Ding Y., Li Y., Peng L., Byon H. R., Goodenough J. B., Yu G. (2015). Chem. Soc. Rev..

[cit7] Winter M., Brodd R. J. (2004). Chem. Rev..

[cit8] Das A., Li D., Williams D., Greenwood D. (2018). World Electric Vehicle Journal.

[cit9] Lichchhavi Lee H., Ohshita Y., Singh A. K., Shirage P. M. (2021). Langmuir.

[cit10] Verma M., Sinha L., Shirage P. M. (2021). J. Mater. Sci.: Mater. Electron..

[cit11] Costa C., Ribelles J. G., Lanceros-Méndez S., Appetecchi G., Scrosati B. (2014). J. Power Sources.

[cit12] Manthiram A., Yu X., Wang S. (2017). Nat. Rev. Mater..

[cit13] Potphode D. D., Sinha L., Shirage P. M. (2019). Appl. Surf. Sci..

[cit14] Zhang W., Tu Z., Qian J., Choudhury S., Archer L. A., Lu Y. (2018). Small.

[cit15] Luo X., Wang J., Dooner M., Clarke J. (2015). Appl. Energy.

[cit16] Zheng J.-S., Zhang L., Shellikeri A., Cao W., Wu Q., Zheng J. P. (2017). Sci. Rep..

[cit17] Vaalma C., Buchholz D., Weil M., Passerini S. (2018). Nat. Rev. Mater..

[cit18] Nayak P. K., Yang L., Brehm W., Adelhelm P. (2018). Angew. Chem., Int. Ed..

[cit19] Nishi Y. (2001). Chem. Rec..

[cit20] Slater M. D., Kim D., Lee E., Johnson C. S. (2013). Adv. Funct. Mater..

[cit21] Kim Y., Ha K. H., Oh S. M., Lee K. T. (2014). Chem.–Eur. J..

[cit22] Abraham K. (1982). Solid State Ionics.

[cit23] Yoshino A. (2012). Angew. Chem., Int. Ed..

[cit24] Xiao J., Li X., Tang K., Wang D., Long M., Gao H., Chen W., Liu C., Liu H., Wang G. (2021). Mater. Chem. Front..

[cit25] Lichchhavi, Lee H., Ohshita Y., Singh A. K., Shirage P. M. (2021). Langmuir.

[cit26] Thackeray M. M., Kang S.-H., Johnson C. S., Vaughey J. T., Benedek R., Hackney S. (2007). J. Mater. Chem..

[cit27] Li W. J., Han C., Wang W., Gebert F., Chou S. L., Liu H. K., Zhang X., Dou S. X. (2017). Adv. Energy Mater..

[cit28] Liu Q., Hu Z., Chen M., Zou C., Jin H., Wang S., Chou S. L., Liu Y., Dou S. X. (2020). Adv. Funct. Mater..

[cit29] Bhojane P., Sinha L., Goutam U. K., Shirage P. M. (2019). Electrochim. Acta.

[cit30] Delmas C., Fouassier C., Hagenmuller P. (1980). Physica B+C.

[cit31] Yabuuchi N., Kubota K., Dahbi M., Komaba S. (2014). Chem. Rev..

[cit32] Pan H., Hu Y.-S., Chen L. (2013). Energy Environ. Sci..

[cit33] Zhang Q., Uchaker E., Candelaria S. L., Cao G. (2013). Chem. Soc. Rev..

[cit34] Muñoz-Márquez M. Á., Saurel D., Gómez-Cámer J. L., Casas-Cabanas M., Castillo-Martínez E., Rojo T. (2017). Adv. Energy Mater..

[cit35] Brand M., Gläser S., Geder J., Menacher S., Obpacher S., Jossen A., Quinger D. (2013). World Electric Vehicle Journal.

[cit36] Dell R. and Rand D. A. J., Understanding batteries, Royal Society of Chemistry, 2001

[cit37] Kurzweil P. (2010). J. Power Sources.

[cit38] Zhang S. S. (2007). J. Power Sources.

[cit39] Shi J.-L., Fang L.-F., Li H., Zhang H., Zhu B.-K., Zhu L.-P. (2013). J. Membr. Sci..

[cit40] Song X., Li X., Bai Z., Yan B., Li D., Sun X. (2016). Nano Energy.

[cit41] Liu X., Wang Y., Wang Z., Zhou T., Yu M., Xiu L., Qiu J. (2017). J. Mater. Chem. A.

[cit42] Wang S., Xia L., Yu L., Zhang L., Wang H., Lou X. W. (2016). Adv. Energy Mater..

[cit43] Palanisamy M., Reddy Boddu V. R., Shirage P. M., Pol V. G. (2021). ACS Appl. Mater. Interfaces.

[cit44] Kanwade A., Gupta S., Kankane A., Srivastava A., Yadav S. C., Shirage P. M. (2022). Sustainable Energy Fuels.

[cit45] You Y., Wu X.-L., Yin Y.-X., Guo Y.-G. (2014). Energy Environ. Sci..

[cit46] Masquelier C., Croguennec L. (2013). Chem. Rev..

[cit47] Rana A. K., Kumar Y., Rajput P., Jha S. N., Bhattacharyya D., Shirage P. M. (2017). ACS Appl. Mater. Interfaces.

[cit48] Kumar Y., Shirage P. M. (2017). J. Mater. Sci..

[cit49] Jin J., Liu Y., Pang X., Wang Y., Xing X., Chen J. (2021). Sci. China: Chem..

[cit50] Wang C., Liu L., Zhao S., Liu Y., Yang Y., Yu H., Lee S., Lee G.-H., Kang Y.-M., Liu R. (2021). Nat. Commun..

[cit51] Liu J., Kan W. H., Ling C. D. (2021). J. Power Sources.

[cit52] Yu Y., Ning D., Li Q., Franz A., Zheng L., Zhang N., Ren G., Schumacher G., Liu X. (2021). Energy Storage Mater..

[cit53] Song T., Kendrick E. (2021). J. Phys.: Mater..

[cit54] Clément R. J., Bruce P. G., Grey C. P. (2015). J. Electrochem. Soc..

[cit55] Sharma A., Bhojane P., Rana A. K., Kumar Y., Shirage P. M. (2017). Scr. Mater..

[cit56] Kubota K., Yabuuchi N., Yoshida H., Dahbi M., Komaba S. (2014). MRS Bull..

[cit57] Mariyappan S., Wang Q., Tarascon J. M. (2018). J. Electrochem. Soc..

[cit58] Kubota K., Dahbi M., Hosaka T., Kumakura S., Komaba S. (2018). Chem. Rec..

[cit59] Kubota K., Fujitani N., Yoda Y., Kuroki K., Tokita Y., Komaba S. (2021). J. Mater. Chem. A.

[cit60] Wang J., Zhou Z., Li Y., Li M., Wang F., Yao Q., Wang Z., Zhou H., Deng J. (2019). J. Alloys Compd..

[cit61] Bhojane P., Sen S., Shirage P. M. (2016). Appl. Surf. Sci..

[cit62] Yao H.-R., Wang P.-F., Gong Y., Zhang J., Yu X., Gu L., OuYang C., Yin Y.-X., Hu E., Yang X.-Q. (2017). J. Am. Chem. Soc..

[cit63] Wang Q., Mariyappan S., Vergnet J., Abakumov A. M., Rousse G., Rabuel F., Chakir M., Tarascon J. M. (2019). Adv. Energy Mater..

[cit64] Mariyappan S., Marchandier T., Rabuel F., Iadecola A., Rousse G., Morozov A. V., Abakumov A. M., Tarascon J.-M. (2020). Chem. Mater..

[cit65] Hwang J.-Y., Yu T.-Y., Sun Y.-K. (2018). J. Mater. Chem. A.

[cit66] Wang Y., Feng Z., Cui P., Zhu W., Gong Y., Girard M.-A., Lajoie G., Trottier J., Zhang Q., Gu L. (2021). Nat. Commun..

[cit67] Hwang J.-Y., Myung S.-T., Sun Y.-K. (2018). J. Phys. Chem. C.

[cit68] Leng M., Bi J., Wang W., Xing Z., Yan W., Gao X., Wang J., Liu R. (2020). J. Alloys Compd..

[cit69] Lee I., Oh G., Lee S., Yu T.-Y., Alfaruqi M. H., Mathew V., Sambandam B., Sun Y.-K., Hwang J.-Y., Kim J. (2021). Energy Storage Mater..

[cit70] Meng X., Zhang D., Zhao Z., Li Y., Xu S., Chen L., Wang X., Liu S., Wu Y. (2021). J. Alloys Compd..

[cit71] Lamb J., Jarvis K., Manthiram A. (2022). Small.

[cit72] Ma L. A., Palm R., Nocerino E., Kenji O., Matsubara N., Cottrell S., Yokoyama K., Koda A., Sugiyama J., Sassa Y. (2021). Phys. Chem. Chem. Phys..

[cit73] Guo S., Sun Y., Yi J., Zhu K., Liu P., Zhu Y., Zhu G.-z., Chen M., Ishida M., Zhou H. (2016). NPG Asia Mater..

[cit74] Tapia-Ruiz N., Armstrong A. R., Alptekin H., Amores M. A., Au H., Barker J., Boston R., Brant W. R., Brittain J. M., Chen Y. (2021). J. Phys.: Energy.

[cit75] Li Z.-Y., Gao R., Zhang J., Zhang X., Hu Z., Liu X. (2016). J. Mater. Chem. A.

[cit76] Lee D. H., Xu J., Meng Y. S. (2013). Phys. Chem. Chem. Phys..

[cit77] Li B., Xia D. (2017). Adv. Mater..

[cit78] Yin C., Wan L., Qiu B., Wang F., Jiang W., Cui H., Bai J., Ehrlich S., Wei Z., Liu Z. (2021). Energy Storage Mater..

[cit79] Yu Y., Karayaylali P., Nowak S. H., Giordano L., Gauthier M., Hong W., Kou R., Li Q., Vinson J., Kroll T. (2019). Chem. Mater..

[cit80] Ma C., Alvarado J., Xu J., Clément R. l. J., Kodur M., Tong W., Grey C. P., Meng Y. S. (2017). J. Am. Chem. Soc..

[cit81] Hakim C., Sabi N., Ma L. A., Dahbi M., Brandell D., Edström K., Duda L. C., Saadoune I., Younesi R. (2020). Commun. Chem..

[cit82] Singh G., Tapia-Ruiz N., Lopez del Amo J. M., Maitra U., Somerville J. W., Armstrong A. R., Martinez de Ilarduya J., Rojo T., Bruce P. G. (2016). Chem. Mater..

[cit83] Doubaji S., Valvo M., Saadoune I., Dahbi M., Edström K. (2014). J. Power Sources.

[cit84] Yang L., Li X., Liu J., Xiong S., Ma X., Liu P., Bai J., Xu W., Tang Y., Hu Y.-Y. (2019). J. Am. Chem. Soc..

[cit85] Xie Y., Gabriel E., Fan L., Hwang I., Li X., Zhu H., Ren Y., Sun C., Pipkin J., Dustin M. (2021). Chem. Mater..

[cit86] Jin T., Wang P. F., Wang Q. C., Zhu K., Deng T., Zhang J., Zhang W., Yang X. Q., Jiao L., Wang C. (2020). Angew. Chem..

[cit87] Liu Z., Shen J., Feng S., Huang Y., Wu D., Li F., Zhu Y., Gu M., Liu Q., Liu J. (2021). Angew. Chem..

[cit88] Li C., Li A., Li M., Xiong P., Liu Y., Cheng M., Geng D., Xu Y. (2022). ACS Appl. Mater. Interfaces.

[cit89] Cheng Z., Zhao B., Guo Y. J., Yu L., Yuan B., Hua W., Yin Y. X., Xu S., Xiao B., Han X. (2022). Adv. Energy Mater..

[cit90] Wang K., Wu Z.-G., Melinte G., Yang Z.-G., Sarkar A., Hua W., Mu X., Yin Z.-W., Li J.-T., Guo X.-D. (2021). J. Mater. Chem. A.

[cit91] Liu Q., Hu Z., Chen M., Zou C., Jin H., Wang S., Chou S.-L., Liu Y., Dou S.-X. (2020). Adv. Funct. Mater..

[cit92] Berthelot R., Carlier D., Delmas C. (2011). Nat. Mater..

[cit93] Biecher Y., Baux A., Fauth F. o., Delmas C., Goward G. R., Carlier D. (2022). Chem. Mater..

[cit94] Zhang R., Lu Z., Yang Y., Shi W. (2018). Curr. Appl. Phys..

[cit95] Sabi N., Sarapulova A., Indris S., Dsoke S., Zhao Z., Dahbi M., Ehrenberg H., Saadoune I. (2019). ChemElectroChem.

[cit96] VN M. O. N. M. M., Dall'Agnese Y. H. M. (2013). Nat. Commun..

[cit97] Fu Z., Wang N., Legut D., Si C., Zhang Q., Du S., Germann T. C., Francisco J. S., Zhang R. (2019). Chem. Rev..

[cit98] Zhang W., Sun X., Tang Y., Xia H., Zeng Y., Qiao L., Zhu Z., Lv Z., Zhang Y., Ge X. (2019). J. Am. Chem. Soc..

[cit99] Ye M., Chen C., Zhang N., Wen X., Guo W., Lin C. (2014). Adv. Energy Mater..

[cit100] Li Q., Guo S., Zhu K., Jiang K., Zhang X., He P., Zhou H. (2017). Adv. Energy Mater..

[cit101] He X., Wang J., Qiu B., Paillard E., Ma C., Cao X., Liu H., Stan M. C., Liu H., Gallash T. (2016). Nano Energy.

[cit102] Tolganbek N., Yerkinbekova Y., Kalybekkyzy S., Bakenov Z., Mentbayeva A. (2021). J. Alloys Compd..

[cit103] Quinzeni I., Fujii K., Bini M., Yashima M., Tealdi C. (2022). Mater. Adv..

[cit104] Cai X., Xu Y., Meng L., Wei X., Xiong F., Xiong T., An Q. (2020). J. Alloys Compd..

[cit105] Li R., Liu Y., Wang Z., Li J. (2019). Electrochim. Acta.

[cit106] Gao R.-M., Zheng Z.-J., Wang P.-F., Wang C.-Y., Ye H., Cao F.-F. (2020). Energy Storage Mater..

[cit107] De La Llave E., Talaie E., Levi E., Nayak P. K., Dixit M., Rao P. T., Hartmann P., Chesneau F., Major D. T., Greenstein M. (2016). Chem. Mater..

[cit108] Chagas L. G., Buchholz D., Wu L., Vortmann B., Passerini S. (2014). J. Power Sources.

[cit109] Hwang J. Y., Kim J., Yu T. Y., Sun Y. K. (2019). Adv. Energy Mater..

[cit110] Wen Y., Fan J., Shi C., Dai P., Hong Y., Wang R., Wu L., Zhou Z., Li J., Huang L. (2019). Nano Energy.

[cit111] Xiao Y., Zhu Y. F., Yao H. R., Wang P. F., Zhang X. D., Li H., Yang X., Gu L., Li Y. C., Wang T. (2019). Adv. Energy Mater..

[cit112] Oh S.-M., Myung S.-T., Hwang J.-Y., Scrosati B., Amine K., Sun Y.-K. (2014). Chem. Mater..

[cit113] Sun L., Xie Y., Liao X. Z., Wang H., Tan G., Chen Z., Ren Y., Gim J., Tang W., He Y. S. (2018). Small.

[cit114] Yang J., Tang M., Liu H., Chen X., Xu Z., Huang J., Su Q., Xia Y. (2019). Small.

[cit115] Xu J., Han Z., Jiang K., Bai P., Liang Y., Zhang X., Wang P., Guo S., Zhou H. (2020). Small.

[cit116] Zhou D., Huang W., Lv X., Zhao F. (2019). J. Power Sources.

[cit117] Bianchini M., Gonzalo E., Drewett N. E., Ortiz-Vitoriano N., del Amo J. M. L., Bonilla F. J., Acebedo B., Rojo T. (2018). J. Mater. Chem. A.

[cit118] Zhou Y.-N., Wang P.-F., Niu Y.-B., Li Q., Yu X., Yin Y.-X., Xu S., Guo Y.-G. (2019). Nano Energy.

[cit119] Chen X., Zhou X., Hu M., Liang J., Wu D., Wei J., Zhou Z. (2015). J. Mater. Chem. A.

[cit120] Wang D., Chen H., Zheng X., Qiu L., Qu J., Wu Z., Zhong Y., Xiang W., Zhong B., Guo X. (2019). ChemElectroChem.

[cit121] Kaliyappan K., Or T., Deng Y. P., Hu Y., Bai Z., Chen Z. (2020). Adv. Funct. Mater..

[cit122] Zhang X., Guo S., Liu P., Li Q., Xu S., Liu Y., Jiang K., He P., Chen M., Wang P. (2019). Adv. Energy Mater..

[cit123] Choi J. U., Park Y. J., Jo J. H., Kuo L.-Y., Kaghazchi P., Myung S.-T. (2018). ACS Appl. Mater. Interfaces.

[cit124] Wang P. F., Yao H. R., Liu X. Y., Zhang J. N., Gu L., Yu X. Q., Yin Y. X., Guo Y. G. (2017). Adv. Mater..

[cit125] Han M. H., Gonzalo E., Sharma N., López del Amo J. M., Armand M., Avdeev M., Saiz Garitaonandia J. J., Rojo T. (2016). Chem. Mater..

[cit126] Zheng W., Liu Q., Wang Z., Wu Z., Gu S., Cao L., Zhang K., Fransaer J., Lu Z. (2020). Energy Storage Mater..

[cit127] Zhang X., Qiao Y., Guo S., Jiang K., Xu S., Xu H., Wang P., He P., Zhou H. (2019). Adv. Mater..

[cit128] Liu X., Zuo W., Zheng B., Xiang Y., Zhou K., Xiao Z., Shan P., Shi J., Li Q., Zhong G. (2019). Angew. Chem., Int. Ed..

[cit129] Konarov A., Choi J. U., Bakenov Z., Myung S.-T. (2018). J. Mater. Chem. A.

[cit130] Hasa I., Passerini S., Hassoun J. (2017). J. Mater. Chem. A.

[cit131] Wang H., Liao X.-Z., Yang Y., Yan X., He Y.-S., Ma Z.-F. (2016). J. Electrochem. Soc..

[cit132] Shi Y., Li S., Gao A., Zheng J., Zhang Q., Lu X., Gu L., Cao D. (2019). ACS Appl. Mater. Interfaces.

[cit133] Luo C., Langrock A., Fan X., Liang Y., Wang C. (2017). J. Mater. Chem. A.

[cit134] Vassilaras P., Ma X., Li X., Ceder G. (2012). J. Electrochem. Soc..

[cit135] Choi J. U., Jo J. H., Park Y. J., Lee K. S., Myung S. T. (2020). Adv. Energy Mater..

[cit136] Shao Y., Tang Z.-f., Liao J.-y., Chen C.-h. (2018). Chin. J. Chem. Phys..

[cit137] Fang T., Guo S., Jiang K., Zhang X., Wang D., Feng Y., Zhang X., Wang P., He P., Zhou H. (2019). Small Methods.

[cit138] De Boisse B. M., Cheng J.-H., Carlier D., Guignard M., Pan C.-J., Bordere S., Filimonov D., Drathen C., Suard E., Hwang B.-J. (2015). J. Mater. Chem. A.

[cit139] Xiao J., Zhang F., Tang K., Li X., Wang D., Wang Y., Liu H., Wu M., Wang G. (2019). ACS Cent. Sci..

[cit140] Tripathi A., Rudola A., Gajjela S. R., Xi S., Balaya P. (2019). J. Mater. Chem. A.

[cit141] Jiang K., Zhang X., Li H., Zhang X., He P., Guo S., Zhou H. (2019). ACS Appl. Mater. Interfaces.

[cit142] Mu L., Xu S., Li Y., Hu Y. S., Li H., Chen L., Huang X. (2015). Adv. Mater..

[cit143] Konarov A., Kim H. J., Voronina N., Bakenov Z., Myung S.-T. (2019). ACS Appl. Mater. Interfaces.

[cit144] Linqin M., Xinguo Q., Yongsheng H., Hong L., Liquan C., Xuejie H. (2016). Energy Storage Sci. Technol..

[cit145] Wang Y., Hu G., Peng Z., Cao Y., Lai X., Qi X., Gan Z., Li W., Luo Z., Du K. (2018). J. Power Sources.

[cit146] Cao M.-H., Shadike Z., Zhou Y.-N., Fu Z.-W. (2019). Electrochim.
Acta.

[cit147] Wang Q. C., Hu E., Pan Y., Xiao N., Hong F., Fu Z. W., Wu X. J., Bak S. M., Yang X. Q., Zhou Y. N. (2017). Adv. Sci..

[cit148] Cao M.-H., Wang Y., Shadike Z., Yue J.-L., Hu E., Bak S.-M., Zhou Y.-N., Yang X.-Q., Fu Z.-W. (2017). J. Mater. Chem. A.

[cit149] Zhang C., Gao R., Zheng L., Hao Y., Liu X. (2018). ACS Appl. Mater. Interfaces.

[cit150] Kaliyappan K., Xaio W., Sham T. K., Sun X. (2018). Adv. Funct. Mater..

[cit151] Yue J.-L., Zhou Y.-N., Yu X., Bak S.-M., Yang X.-Q., Fu Z.-W. (2015). J. Mater. Chem. A.

[cit152] Zhang J., Wang W., Wang W., Wang S., Li B. (2019). ACS Appl. Mater. Interfaces.

[cit153] Wang J. E., Han W. H., Chang K. J., Jung Y. H., Kim D. K. (2018). J. Mater. Chem. A.

[cit154] Wang K., Wan H., Yan P., Chen X., Fu J., Liu Z., Deng H., Gao F., Sui M. (2019). Adv. Mater..

[cit155] Deng J., Luo W. B., Lu X., Yao Q., Wang Z., Liu H. K., Zhou H., Dou S. X. (2018). Adv. Energy Mater..

[cit156] Zhang X.-H., Pang W.-L., Wan F., Guo J.-Z., Lü H.-Y., Li J.-Y., Xing Y.-M., Zhang J.-P., Wu X.-L. (2016). ACS Appl. Mater. Interfaces.

[cit157] Liu Y., Shen Q., Zhao X., Zhang J., Liu X., Wang T., Zhang N., Jiao L., Chen J., Fan L. Z. (2020). Adv. Funct. Mater..

[cit158] You Y., Xin S., Asl H. Y., Li W., Wang P.-F., Guo Y.-G., Manthiram A. (2018). Chem.

[cit159] Li C., Zhao C., Hu B., Tong W., Shen M., Hu B. (2020). Chem. Mater..

[cit160] Zhang L., Yuan T., Soule L., Sun H., Pang Y., Yang J., Zheng S. (2020). ACS Appl. Energy Mater..

[cit161] Zheng S., Zhong G., McDonald M. J., Gong Z., Liu R., Wen W., Yang C., Yang Y. (2016). J. Mater. Chem. A.

[cit162] Wang P.-F., You Y., Yin Y.-X., Guo Y.-G. (2016). J. Mater. Chem. A.

[cit163] Boddu V. R. R., Palanisamy M., Sinha L., Yadav S. C., Pol V. G., Shirage P. M. (2021). Sustainable Energy Fuels.

[cit164] Dall'Asta V., Buchholz D., Chagas L. G., Dou X., Ferrara C., Quartarone E., Tealdi C., Passerini S. (2017). ACS Appl. Mater. Interfaces.

[cit165] Liu Q., Hu Z., Chen M., Gu Q., Dou Y., Sun Z., Chou S., Dou S. X. (2017). ACS Appl. Mater. Interfaces.

[cit166] Fu B., Su Y., Yu J., Xie S., Li J. (2019). Electrochim. Acta.

[cit167] Dai K., Mao J., Song X., Battaglia V., Liu G. (2015). J. Power Sources.

[cit168] Fu B., Zhou X., Wang Y. (2016). J. Power Sources.

[cit169] Hosono E., Saito T., Hoshino J., Okubo M., Saito Y., Nishio-Hamane D., Kudo T., Zhou H. (2012). J. Power Sources.

[cit170] Sheng N., Han C.-g., Lei Y., Zhu C. (2018). Electrochim. Acta.

[cit171] Zhang D., Shi W.-j., Yan Y.-w., Xu S.-d., Chen L., Wang X.-m., Liu S.-b. (2017). Electrochim. Acta.

[cit172] Qiao R., Dai K., Mao J., Weng T.-C., Sokaras D., Nordlund D., Song X., Battaglia V. S., Hussain Z., Liu G. (2015). Nano Energy.

[cit173] Pahari D., Puravankara S. (2020). J. Power Sources.

[cit174] Yao H. R., Wang P. F., Wang Y., Yu X., Yin Y. X., Guo Y. G. (2017). Adv. Energy Mater..

[cit175] Wang L., Wang J., Zhang X., Ren Y., Zuo P., Yin G., Wang J. (2017). Nano Energy.

[cit176] Wang Y., Wang L., Zhu H., Chu J., Fang Y., Wu L., Huang L., Ren Y., Sun C. J., Liu Q. (2020). Adv. Funct. Mater..

[cit177] Komaba S., Yabuuchi N., Nakayama T., Ogata A., Ishikawa T., Nakai I. (2012). Inorg. Chem..

[cit178] Wang K., Yan P., Sui M. (2018). Nano Energy.

[cit179] Wang L., Wang J., Guo F., Ma L., Ren Y., Wu T., Zuo P., Yin G., Wang J. (2018). Nano Energy.

[cit180] Liu Q., Hu Z., Chen M., Zou C., Jin H., Wang S., Gu Q., Chou S. (2019). J. Mater. Chem. A.

[cit181] Wang L., Sun Y.-G., Hu L.-L., Piao J.-Y., Guo J., Manthiram A., Ma J., Cao A.-M. (2017). J. Mater. Chem. A.

[cit182] Zuo W., Qiu J., Liu X., Ren F., Liu H., He H., Luo C., Li J., Ortiz G. F., Duan H. (2020). Nat. Commun..

[cit183] Liu Q., Hu Z., Chen M., Zou C., Jin H., Wang S., Chou S. L., Dou S. X. (2019). Small.

[cit184] Yabuuchi N., Kajiyama M., Iwatate J., Nishikawa H., Hitomi S., Okuyama R., Usui R., Yamada Y., Komaba S. (2012). Nat. Mater..

[cit185] Aragón M. J., Lavela P., Ortiz G., Alcántara R., Tirado J. L. (2017). J. Alloys Compd..

[cit186] Bhojane P., Sharma A., Pusty M., Kumar Y., Sen S., Shirage P. (2017). J. Nanosci. Nanotechnol..

[cit187] Shi C., Wang L., Chen X., Li J., Wang S., Wang J., Jin H. (2022). Nanoscale Horiz..

[cit188] Shanmugam G., Deshpande U., Sharma A., Shirage P., Bhobe P. (2018). J. Phys. Chem. C.

[cit189] Li Y., Chen M., Liu B., Zhang Y., Liang X., Xia X. (2020). Adv. Energy Mater..

[cit190] Xu H., Yan Q., Yao W., Lee C.-S., Tang Y. (2022). Small Struct..

[cit191] Xi K., Chu S., Zhang X., Zhang X., Zhang H., Xu H., Bian J., Fang T., Guo S., Liu P. (2020). Nano Energy.

[cit192] Shi W.-J., Zhang D., Meng X.-M., Bao C.-X., Xu S.-D., Chen L., Wang X.-M., Liu S.-B., Wu Y.-C. (2020). ACS Appl. Mater. Interfaces.

[cit193] Verma M., Yadav R., Sinha L., Mali S. S., Hong C. K., Shirage P. M. (2018). RSC Adv..

[cit194] Chen H., Wu Z., Zhong Y., Chen T., Liu X., Qu J., Xiang W., Li J., Chen X., Guo X. (2019). Electrochim. Acta.

[cit195] Lin X., Sun Y., Liu Y., Jiang K., Cao A. (2021). Energy Fuels.

[cit196] Gu Z.-Y., Guo J.-Z., Sun Z.-H., Zhao X.-X., Li W.-H., Yang X., Liang H.-J., Zhao C.-D., Wu X.-L. (2020). Sci. Bull..

[cit197] Lu D., Yao Z., Li Y., Zhong Y., Wang X., Xie D., Xia X., Gu C., Tu J. (2020). J. Colloid Interface Sci..

[cit198] Jo J. H., Choi J. U., Konarov A., Yashiro H., Yuan S., Shi L., Sun Y. K., Myung S. T. (2018). Adv. Funct. Mater..

[cit199] Zhang J., Zhou X., Wang Y., Qian J., Zhong F., Feng X., Chen W., Ai X., Yang H., Cao Y. (2019). Small.

[cit200] Zhang J., Chen Z., Ai Q., Terlier T., Hao F., Liang Y., Guo H., Lou J., Yao Y. (2021). Joule.

[cit201] Kaliyappan K., Xiao W., Adair K. R., Sham T.-K., Sun X. (2018). ACS Omega.

