# Correction: Transition metal oxides as a cathode for indispensable Na-ion batteries

**DOI:** 10.1039/d2ra90080g

**Published:** 2022-08-30

**Authors:** Archana Kanwade, Sheetal Gupta, Akash Kankane, Manish Kumar Tiwari, Abhishek Srivastava, Jena Akash Kumar Satrughna, Subhash Chand Yadav, Parasharam M. Shirage

**Affiliations:** Department of Metallurgy Engineering and Materials Science, Indian Institute of Technology Indore 453552 India pmshirage@iiti.ac.in paras.shirage@gmail.com; Department of Physics, Indian Institute of Technology Indore 453552 India

## Abstract

Correction for ‘Transition metal oxides as a cathode for indispensable Na-ion batteries’ by Archana Kanwade *et al.*, *RSC Adv.*, 2022, **12**, 23284–23310, https://doi.org/10.1039/d2ra03601k.

The authors regret that the author list was shown incorrectly in the original article. The correct author list is as shown below:

Sheetal Gupta†^a^, Archana Kanwade†^a^, Akash Kankane^a^, Manish Kumar Tiwari^a^, Abhishek Srivastava^a^, Jena Akash Kumar Satrughna^b^, Subhash Chand Yadav^a^ and Parasharam M. Shirage*^a^

^a^Department of Metallurgy Engineering and Materials Science, Indian Institute of Technology, Indore 453552, India, E-mail: pmshirage@iiti.ac.in, paras.shirage@gmail.com

^b^Department of Physics, Indian Institute of Technology, Indore 453552, India

† Equal contributions.

Additionally, the authors regret that the photos of Ms Archana Kanwade and Ms Sheetal Gupta are incorrectly displayed. The correct photos are shown below:

(1) The photo of Ms Sheetal Gupta is as follows:
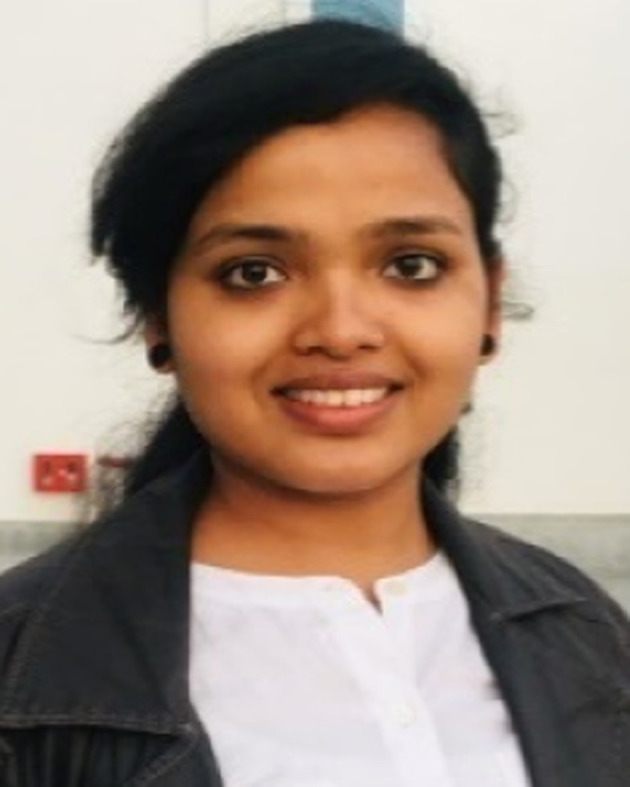


(2) The photo of Ms Archana Kanwade is as follows:
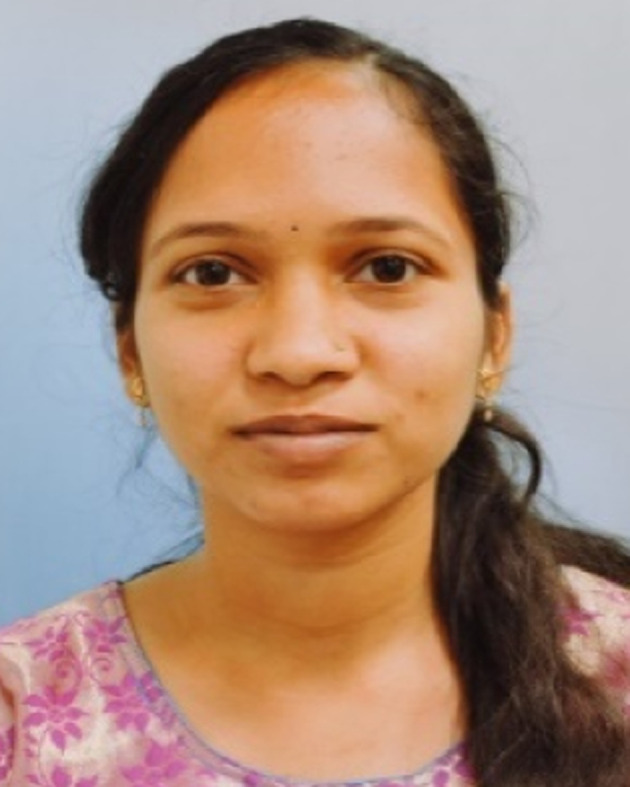


The Royal Society of Chemistry apologises for these errors and any consequent inconvenience to authors and readers.

## Supplementary Material

